# PARAQUAT TOLERANCE3 Is an E3 Ligase That Switches off Activated Oxidative Response by Targeting Histone-Modifying PROTEIN METHYLTRANSFERASE4b

**DOI:** 10.1371/journal.pgen.1006332

**Published:** 2016-09-27

**Authors:** Chao Luo, Xiao-Teng Cai, Jin Du, Tao-Lan Zhao, Peng-Fei Wang, Ping-Xia Zhao, Rui Liu, Qi Xie, Xiao-Feng Cao, Cheng-Bin Xiang

**Affiliations:** 1 School of Life Sciences, University of Science and Technology of China, Hefei, Anhui Province, China; 2 State Key Laboratory of Plant Genomics and National Center for Plant Gene Research, Institute of Genetics and Developmental Biology, Chinese Academy of Science, Beijing, China; University Of Leeds, UNITED KINGDOM

## Abstract

Oxidative stress is unavoidable for aerobic organisms. When abiotic and biotic stresses are encountered, oxidative damage could occur in cells. To avoid this damage, defense mechanisms must be timely and efficiently modulated. While the response to oxidative stress has been extensively studied in plants, little is known about how the activated response is switched off when oxidative stress is diminished. By studying Arabidopsis mutant *paraquat tolerance3*, we identified the genetic locus *PARAQUAT TOLERANCE3* (*PQT3*) as a major negative regulator of oxidative stress tolerance. *PQT3*, encoding an E3 ubiquitin ligase, is rapidly down-regulated by oxidative stress. PQT3 has E3 ubiquitin ligase activity in ubiquitination assay. Subsequently, we identified PRMT4b as a PQT3-interacting protein. By histone methylation, PRMT4b upregulates the expression of *APX1* and *GPX1*, encoding two key enzymes against oxidative stress. On the other hand, PRMT4b is recognized by PQT3 for targeted degradation via 26S proteasome. Therefore, we have identified PQT3 as an E3 ligase that acts as a negative regulator of activated response to oxidative stress and found that histone modification by PRMT4b at *APX1* and *GPX1* loci plays an important role in oxidative stress tolerance.

## Author Summary

Oxidative stress is a major stress in plant cells when biotic and abiotic stresses are imposed. While the response to oxidative stress has been extensively studied, little is known about how the activated response is switched off when oxidative stress is diminished. By studying Arabidopsis mutant *paraquat tolerance3*, we identified the genetic locus *PARAQUAT TOLERANCE3* (*PQT3*) as a major negative regulator of oxidative tolerance. *PQT3* encodes an E3 ubiquitin ligase and is rapidly down-regulated by oxidative stress. Subsequently, we identified PRMT4b as a PQT3-interacting protein. PQT3 was demonstrated to recognize PRMT4b for targeted degradation via 26S proteasome. By histone methylation, PRMT4b may regulate the expression of *APX1* and *GPX1*, encoding two key enzymes against oxidative stress. Therefore, we have identified PQT3 as an E3 ubiquitin ligase that turns off the activated response to oxidative stress. Our study provides new insights into the post-translational regulation of plant oxidative stress response and ROS signaling.

## Introduction

Sessile plants cannot avoid harsh living conditions such as drought, salinity, cold and hot temperature. These stresses alter the normal cell homeostasis and increase the generation of reactive oxygen species (ROS) [1]. ROS could also be generated by paraquat, one widely used herbicide [2]. Accumulation of ROS directly destroys biological membranes and macromolecules, accelerates cell senescence, induces irreversible damages to cells and even leads to cell death [3]. The level of ROS is increased sharply under stress conditions [1, 4–9]. Two protection systems, enzymatic and non-enzymatic, have evolved to scavenge ROS and protect plant cells from oxidative damage. Enzymatic system mainly includes the ascorbate peroxidase (APX), glutathione peroxidase (GPX), catalase (CAT), superoxide dismutase (SOD), and peroxiredoxin Q (PRXQ) [8, 10, 11]. The transcript level and activity of antioxidant enzymes correlate with paraquat tolerance [12].

The activation of oxidative response involves many layers of regulations [8, 13]. Little is known about the regulation by histone methylation of the genes involved in oxidative stress response. Histone methylation plays important roles in the plant development and growth as well as in some stress responses [14–18]. The methylation marks are written on lysines or arginines respectively by protein arginine methyltransferases (PRMTs) and histone lysine methyltransferases (HKMTs). In Arabidopsis, nine PRMTs are found in the genome [14]. It has been reported that PRMTs are involved in salt stress responses, flowering time as well as circadian cycle [19, 20]. Two different types of PRMTs catalyze asymmetric di-methylation (ADMA) and symmetric di-methylation (SDMA) on the Arg residues, respectively [17, 21]. The *prmt5* mutant has decreased level of histone H4 Arg3-SDMA, leading to enhanced drought tolerance [22]. A pair of PROTEIN ARGININE METHYLTRANSFERASE4 (PRMT4) homologs, AtPRMT4a and AtPRMT4b, is required for the asymmetrical di-methylation of Arg-2, Arg-17, and Arg-26 in histone H3 [14]. The *prmt4aprmt4b* double mutants are sensitive to salt stress [20]. Protein arginine methylation plays essential roles in diverse biological processes, such as RNA processing and transcriptional regulation [21, 23].

Oxidative stress could be perceived by multiple mechanisms, including sensor or cellular receptor. The perception by receptors results in the activation of Ca^2+^–calmodulin and mitogen-activated protein kinase (MAPK) cascade signaling transduction pathway. The activation or suppression of different transcription factors regulates a variety of defense pathway subsequently, such as ROS-scavenging, heat-shock proteins (HSPs), and photosynthesis [8, 10, 13]. Several paraquat tolerance mutants have been analyzed in Arabidopsis [24]. While much attention has been paid to how plants respond to oxidative stress, we know little about how plants switch off the activated responses when stress is diminished. A common regulatory mechanism is to control the protein level of the stress responsive factors. The most studied mechanism of protein degradation is the ubiquitin/26S proteasome system [25]. The ubiquitin/26S proteasome pathway is involved in different regulatory processes of eukaryotic cells, as it can rapidly eliminate the specific proteins in the cell [25–27]. Ubiquitin, containing 76 amino acids, could be attached to the target protein under the action of three different enzymes [27]. The ubiquitin system can identify and modify many intracellular proteins, such as proteins involved in signal transduction, transcription factors, and receptors on cell surface, to participate in the regulation of physiological processes [28]. The target proteins with ubiquitin have different fates. For monoubiquitination, one lysine residue of substrate is modified by a single ubiquitin. If several individual lysine residues of target protein are attached with single ubiquitin respectively, the protein modification is named multiubiquitination. Both mono- and multi-ubiquitination could affect protein activity and intracellular localization [29–31]. For polyubiquitination, one ubiquitin is attached to lysine residue of substrate firstly. The C-terminal glycine of next ubiquitin is linked with lysine residue of the preceding ubiquitin to form polyubiquitin chain subsequently [32]. As ubiquitin contains seven lysines, polyubiquitin chains are divided into different types according to different linkages between two adjacent ubiquitins [33]. Proteins with Lys48-Linkage polyubiquitination could be recognized and degraded by the 26S proteasome [34]. It has also been reported that Lys29-Linkage polyubiquitination involved in proteasome-dependent degradation [35]. In addition, Lys63-Linkage polyubiquitination plays roles in endocytosis, repair of DNA damage, protein synthesis and signal transduction [36, 37].

In the ubiquitin degradation process, E3 played a crucial role. E3 is responsible for specific recognition of substrate protein and accurate positioning of the binding site between substrate protein and ubiquitin [38]. In *Arabidopsis thaliana*, the genes encoded the subunits of E3 ubiquitin ligases make up approximately 90 percent of about 1400 genes encoded the components of ubiquitin/26S proteasome pathway. [26, 39]. The large and diverse family of plant E3 ubiquitin ligases can be divided into HECT domain- and RING/U-box domain-containing E3 ubiquitin ligases [25]. The HECT family is relatively small compared with the RING domain-containing family that contains several hundreds of proteins and can be further divided into single subunit RING/U-box E3 ligases and multi subunit RING E3 ligases [26, 40]. The large number of E3 ubiquitin ligases in higher plants indicates their important regulatory roles in diverse biological processes [41].

Several RING E3 ligases play positive roles in ABA-mediated drought tolerance [42–45]. In addition, overexpression of RING E3 ligases Rma1 inhibited the trafficking of aquaporin PIP2;1 and promote protein degradation of PIP2;1 to enhance drought tolerance [46]. A few E3 ligases have also been identified to turn off the activated stress responses [47]. HOS1, as a RING E3 ligase, acts as a negative regulator in cold tolerance. HOS1 inhibits the expression of CBFs and downstream cold-responsive genes through the degradation of ICE1 in ubiquitin-proteasome pathway [48–50]. Both PUB22 and PUB23, as E3 ligases contained U-box, play roles as negative regulators in drought tolerance. They could recognize and degrade RPN12a, a subunit of 19S regulatory particle belonged to 26S proteasome, to affect Arabidopsis drought tolerance [51]. The negative function of PUB18 and PUB19 in ABA-mediated drought tolerance has also been reported [52]. The drought-induced AtERF53 could be degraded by RING E3 Ligase RGLG1 and RGLG2 that acts as negative regulators in drought tolerance [53]. Overexpression of salt-induced RING E3 ligase OsSIRP1 reduced salt tolerance in Arabidopsis [54]. In addition, several substrate receptors of CUL4 E3 ligases, DWAs, regulate ABA responses negatively [55, 56]. The function of an enormous number of E3 ligases still remains to be identified. Here we report a novel negative regulator of oxidative stress response by PARAQUAT TOLERANCE3 (PQT3) in Arabidopsis. We identified a paraquat tolerant mutant, ***p****ara****q****uat* ***t****olerance3* (*pqt3*), and cloned the gene *PQT3* that encodes an E3 ligase containing RING/U-box domain. The function of *PQT3* was revealed, although *PQT3* (*At4g17410*) had been identified in previous microarray data on salt stress response and cold acclimation [57, 58]. The expression of *APX1* and *GPX1* was up-regulated in *pqt3*, while *PQT3* was down regulated by oxidative stress. PQT3 was able to interact with PRMT4b. PRMT4b may catalyze histone methylation on *APX1* and *GPX1* chromatin and up-regulate their expressions, therefore protect plants from oxidative stress. When oxidative stress is diminished, PQT3 level increases and acts as E3 ubiquitin ligase to specifically target PRMT4b for degradation. Based on our results, PQT3 is a negative regulator that turns off the activated response of oxidative stress.

## Results

### Loss of *At4g17410* confers the paraquat tolerance of *pqt3*

**Fig 1:**
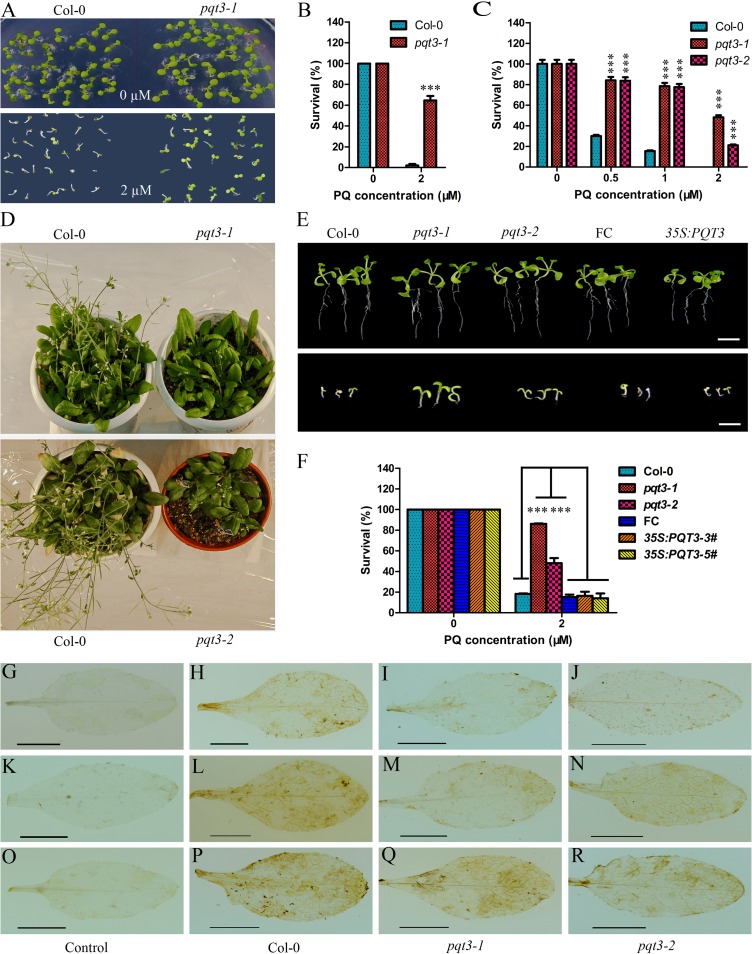
Phenotype of *pqt3-1* and *pqt3-2* mutants. **(A)** Confirmation of paraquat (PQ) tolerant phenotype. 7-day-old seedlings of wild type and *pqt3* mutant were grown on MS medium supplemented with 0 or 2 μM paraquat. **(B)** Survival ratio of wild type and *pqt3* mutant grown in **(A)**. Values are mean ±SD (n = 30 plants, ***P < 0.001). Asterisks indicate Student’s t-test significant differences. **(C)** Multiple mutant alleles analysis of paraquat tolerance for the *At4g17410* locus. Survival ratio of wild type, *pqt3-1* and *pqt3-2* (Salk_065409) grown in the 0, 0.5, 1, and 2 μM paraquat medium was counted. Values are mean ± SD (n = 30 plants, ***P < 0.001). Asterisks indicate Student’s t-test significant differences. **(D)** Late-flowering phenotype of *pqt3-1* and *pqt3-2* compared with wild type. Plants were grown under long day photoperiod (16 h light and 8 h dark). **(E)** The phenotype of 7-day-old *pqt3-1*, wild type, function complementation (FC), *pqt3-2*, and *35Spro*:*PQT3* seedlings under 0 μM (the top picture) or 2 μM (the bottom picture) paraquat treatment. Bar = 0.5 cm. **(F)** The survival ratio of *pqt3-1*, wild type, FC, *pqt3-2*, and *35Spro*:*PQT3* seeds germinated and grown on MS medium containing 0 μM or 2 μM paraquat for 7 days was counted. Values are mean ± SD (n = 30 plants, ***P < 0.001). Asterisks indicate Student’s t-test significant differences. **(G-R)** DAB staining. The leaves of wild type, *pqt3-1*, and *pqt3-2* were treated without **(H-J)** or with 6 μM paraquat for 12 **(L-N)** or 24 h **(P-R)**. 10 mM Na_2_HPO_4_ was used as the negative control to stain the leaves of wild type treated without **(G)** or with 6 μM paraquat for 12 **(K)** or 24 h **(O)**. Bar = 0.5 cm.

The mutant *pqt3* was obtained after screening an activation-tagging library [59]. This library consisting of approximately 55, 000 independent lines was screened for mutants with enhanced tolerance to different stresses [60, 61]. To isolate tolerant mutant to oxidative stress, we germinated seeds on MS medium with 2 μM paraquat. Growing green seedlings were selected as putative mutants and were named *paraquat tolerance* (*pqt*) because of their enhanced tolerance to paraquat. The *pqt3*, one of such mutants, was further characterized and marked as *pqt3-1*. The enhanced oxidative tolerance of *pqt3-1* mutant was confirmed by germinating seeds on MS medium containing 0 or 2 μM paraquat. Based on the observation of green cotyledons, survival ratio were counted. In presence of paraquat, more than 60% *pqt3-1* seeds germinated with green cotyledons but only 2% wild type seeds did, while all seeds of both wild type and *pqt3-1* survived on MS medium without paraquat (Fig 1A and 1B). Genetic analysis showed that the mutation was recessive. All F1 backcross offsprings (*pqt3-1* x wild type) were paraquat sensitive and F2 selfing population showed typical 3:1 segregation ratio (sensitive: resistant; 85:27, χ^2^ = 0.0476). The result suggested that *pqt3-1* mutant may have a more efficient mechanism of ROS scavenge, which was caused by loss-of-function mutation in a single nuclear gene *PQT3* (*At4g17410*).

In *pqt3-1* mutant, a single T-DNA insertion was located in the fourth intron of *At4g17410*(S1A Fig). The exact integration site of the T-DNA right border was 803bp downstream of the ATG initiation codon of *At4g17410*. As a result, the expression of *At4g17410* was completely disrupted as confirmed by RT-PCR analysis (S1B and S1D Fig). The expressions of its neighboring genes, *At4g17390* and *At4g17420*, were not affected (S1B Fig). The *At4g17410* locus includes 13 exons and 12 introns. By prediction, the open reading frame encodes a 91 kD polypeptide composed of 827 amino acids. Based on the conserved RING/U-box domain, this protein is predicted as an E3 ubiquitin ligase.

To further determine whether the loss of *At4g17410* resulted in the enhanced oxidative tolerance of *pqt3-1* mutant, we used another allele of *pqt3*, the T-DNA insertion mutant Salk_065409, which was ordered from Arabidopsis Biological Resource Center (ABRC) and its T-DNA insertion was confirmed by RT-PCR (S1A, S1C and S1D Fig). As the first identified *pqt3* mutant was named as *pqt3-1*, the Salk_065409 was marked as *pqt3-2*. The *pqt3-2* mutant showed similar enhanced oxidative tolerance to paraquat and had high survival ratio under different concentrations of paraquat treatment as *pqt3-1* did (Fig 1C). The survival ratio of *pqt3-1* and *pqt3-2* were 50% and 20%, respectively, under 2 μM paraquat treatment, while none of the wild type seedlings survived under the same condition. In addition, both *pqt3-1* and *pqt3-2* showed a late-flowering phenotype (Fig 1D).

To confirm further, we generated functional complementation (FC) lines and *35Spro*:*PQT3* overexpression lines (S1E and S1F Fig). FC lines and *35Spro*:*PQT3* lines showed similar if not higher paraquat sensitivity to wild type under 2 μM paraquat treatment while the *pqt3-1* and the *pqt3-2* mutants displayed enhanced paraquat tolerance (Fig 1E and 1F). These results indicate that PQT3 is a negative regulator of oxidative stress tolerance and is responsible for the phenotype of *pqt3* mutants.

In addition, we assayed H_2_O_2_ level in the leaf with DAB staining, in which the chemical reaction between hydrogen peroxide and DAB lead to the formation of brown precipitate that indicates hydrogen peroxide distribution and oxidative damage. After 6 μM paraquat treatment for 12 or 24 hours, the result of 3,3′- diaminobenzidine (DAB) staining showed that the brown precipitate in the leaves of the wild type was more than that of *pqt3* mutants (Fig 1G–1R). As several stresses could cause oxidative damage to plants, the sensitivity of *pqt3* mutants to other environmental stresses was analyzed subsequently. The result indicated that *pqt3* mutants have enhanced tolerance to CdCl_2_, mannitol, NaCl, and drought stress (S2 Fig).

### Spatiotemporal expression pattern and protein localization of *PQT3*

**Fig 2:**
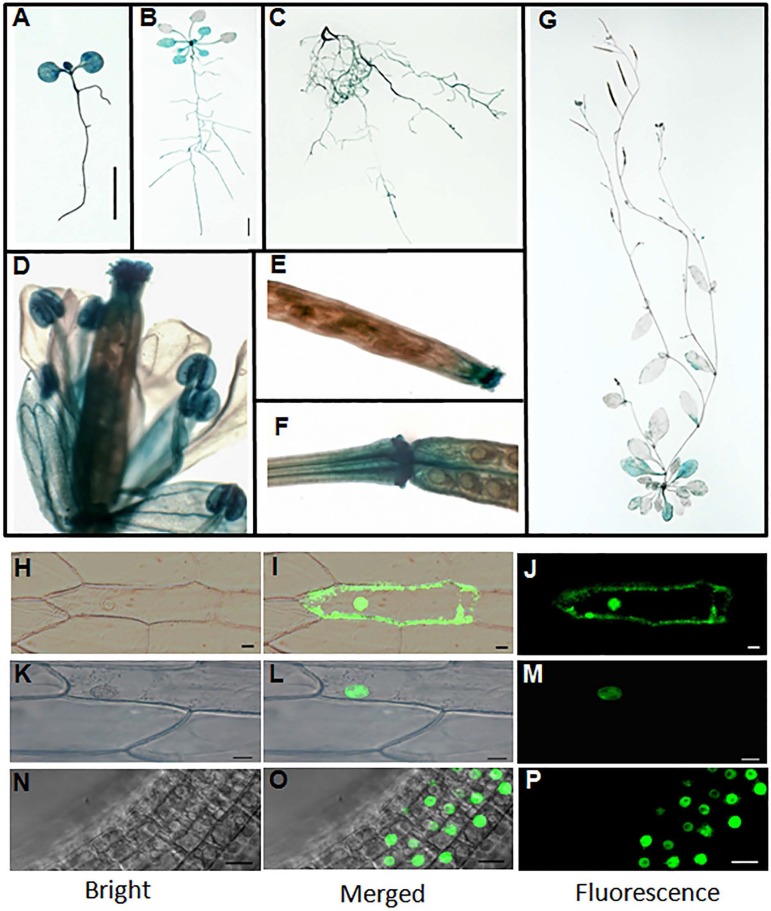
Expression pattern and subcellular localization of PQT3. **(A)** GUS expression in 1-week-old seedling. Transgenic Arabidopsis plants expressing *PQT3pro*:*GUS* were generated and analyzed for GUS expression. Bar = 0.5 cm. **(B)** GUS expression in 3-week-old seedling. Bar = 0.5 cm. **(C)** GUS expression in root tissue of 7-week-old plant. **(D)** GUS expression in flower tissue. **(E and F)** GUS expression in silique tip **(E)** and junction **(F)**. **(G)** GUS expression in 7-week-old adult Arabidopsis. **(H-J)** *35Spro*:*GFP* was transiently expressed in onion epidermal cells as control. The GFP can be observed in both plasma membrane and nucleus. **(K-M)** Nucleus localization of the PQT3-GFP fusion protein in onion epidermal cell was observed. Bar = 20 μm. **(N-P)** The nucleus localization of PQT3-GFP fusion protein in the root tissue of stable transgenic seedlings expressing *PQT3pro*:*PQT3-GFP*. Bar = 20 μm.

To investigate the spatiotemporal pattern of *PQT3* expression, we generated *PQT3pro*:*GUS* reporter lines. GUS staining results showed that *PQT3* was expressed in both shoot and root tissues under normal condition (Fig 2A–2G). GUS expression was detected in the root tissues at all developmental stages we analyzed (Fig 2A–2C). For the 1-week-old seedlings, strong GUS staining was observed in the cotyledons, hypocotyls and root tissues (Fig 2A). For the 3-week-old seedlings, strong GUS staining was also detected in cotyledons, young leaves, and root tissues, but weakly stained in older leaves (Fig 2B). In 7-week-old adult plants, GUS expression was detected in rosette leaves, cauline leaves, the tip and basal junction of siliques, and was significantly higher in the flower petals, stamens and stigma of pistil (Fig 2D–2G).

PQT3 has two predicted nuclear localization signals (NLSs) in the carboxyl terminus (S3 Fig), implicating its nuclear localization. To confirm this, the *35Spro*:*PQT3-GFP* construct was made and transiently expressed in onion epidermal cells. The *35Spro*:*GFP* construct was used as control (Fig 2H–2J). PQT3-GFP signal was indeed detected in the nucleus (Fig 2K–2M).

For further confirmation, we transformed the *PQT3pro*: *PQT3-GFP* fusion construct into the Arabidopsis and obtained transgenic plants. Fluorescent microscopy results showed that GFP signal was accumulated in the nucleus of root cells (Fig 2N–2P), which is in agreement with the presence of two NLSs of the PQT3.

### *PQT3* is rapidly down-regulated by stress treatments

**Fig 3:**
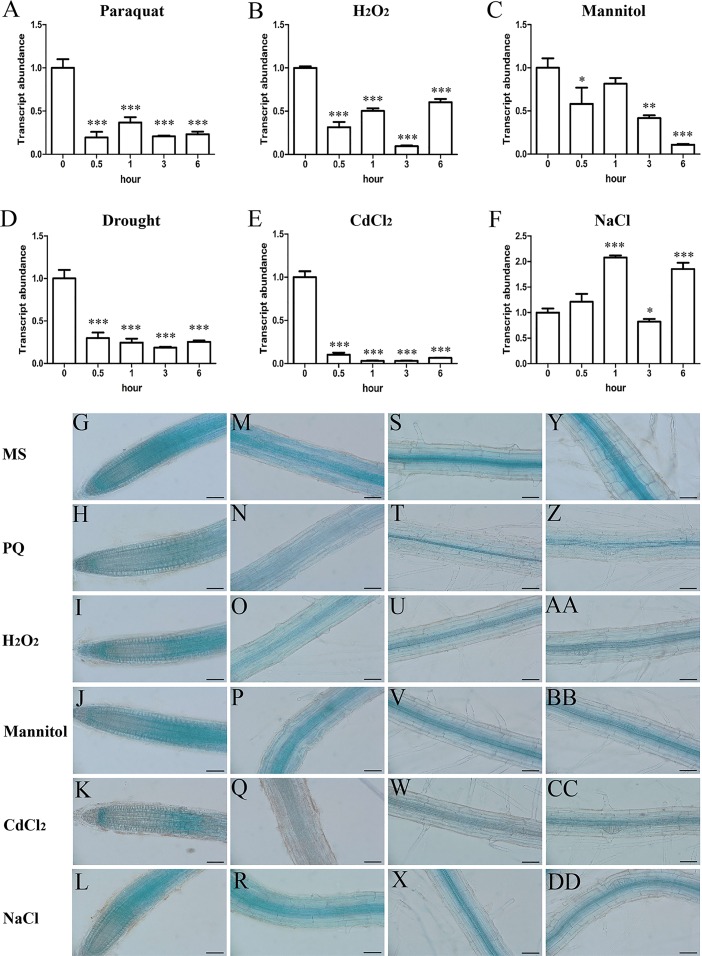
The expression of *PQT3* is rapidly down-regulated by various oxidative stress conditions. **(A)** The transcript level of *PQT3* was down-regulated by paraquat treatment. 1-week-old seedlings were treated with 6 μM paraquat for the indicated times before RNA extraction for quantitative RT-PCR analysis. Values are mean ± SD (n = 3 experiments, ***P < 0.001). Asterisks indicate Student’s t-test significant differences. **(B-F)** The expression of *PQT3* was down-regulated by other stress conditions. 1-week-old seedlings were treated by 10 mM H_2_O_2_ **(B)**, 200 mM mannitol **(C)**, drought **(D)**, 200 mM CdCl_2_ **(E)**, and 150 mM NaCl **(F)** for the indicated times before RNA extraction for quantitative RT-PCR analysis. Values are mean ± SD (n = 3 experiments, *P < 0.05, **P < 0.01, ***P < 0.001). Asterisks indicate Student’s t-test significant differences. **(G-DD)** GUS staining of 7-day-old *PQT3pro*:*GUS* transgenic lines without or with 6 μM paraquat, 10 mM H_2_O_2_, 200 mM mannitol, 200 mM CdCl_2_, 150 mM NaCl treatment for 3 h. GUS expressions were significantly reduced in primary root tip **(G-L)**, root elongation zone **(M-R)**, root maturation zone **(S-X)**, root zone with LRP **(Y-DD)** under stress conditions. Bar = 50 μm.

The expression of *PQT3* was rapidly down-regulated by paraquat treatment and maintained at a low level as long as the paraquat treatment was applied (Fig 3A). This result is supportive for our previous opinion that PQT3 is a negative regulator of plant oxidative tolerance. The suppressed transcript level of *PQT3* was restored to the previous level when PQ stress diminished (S4 Fig). By extrapolation, the expression of *PQT3* could be down-regulated by other stress conditions. Indeed, our results showed that the expression of *PQT3* was down-regulated by H_2_O_2_ (Fig 3B), mannitol (Fig 3C), drought (Fig 3D) and CdCl_2_ (Fig 3E) at the indicated time points. Among these stresses, CdCl_2_ treatment led to the most significant reduction of the expression of *PQT3* (Fig 3E). The expression of *PQT3* could also be down-regulated by NaCl stress at 3h. However, unlike the above results, the expression of *PQT3* was activated by salt treatment at other time points (Fig 3F).

The *PQT3pro*:*GUS* pattern was observed under different stresses subsequently (Fig 3G–3DD). Compared with transgenic seedlings under normal conditions, GUS staining was weaker in the seedlings under paraquat, H_2_O_2_, mannitol, CdCl_2_, and NaCl treatment. The changed *PQT3pro*:*GUS* pattern was consistent with the altered expression of *PQT3* detected by quantitative RT-PCR under different stresses.

### *APX1* and *GPX1* are up-regulated in *pqt3* mutant

**Fig 4:**
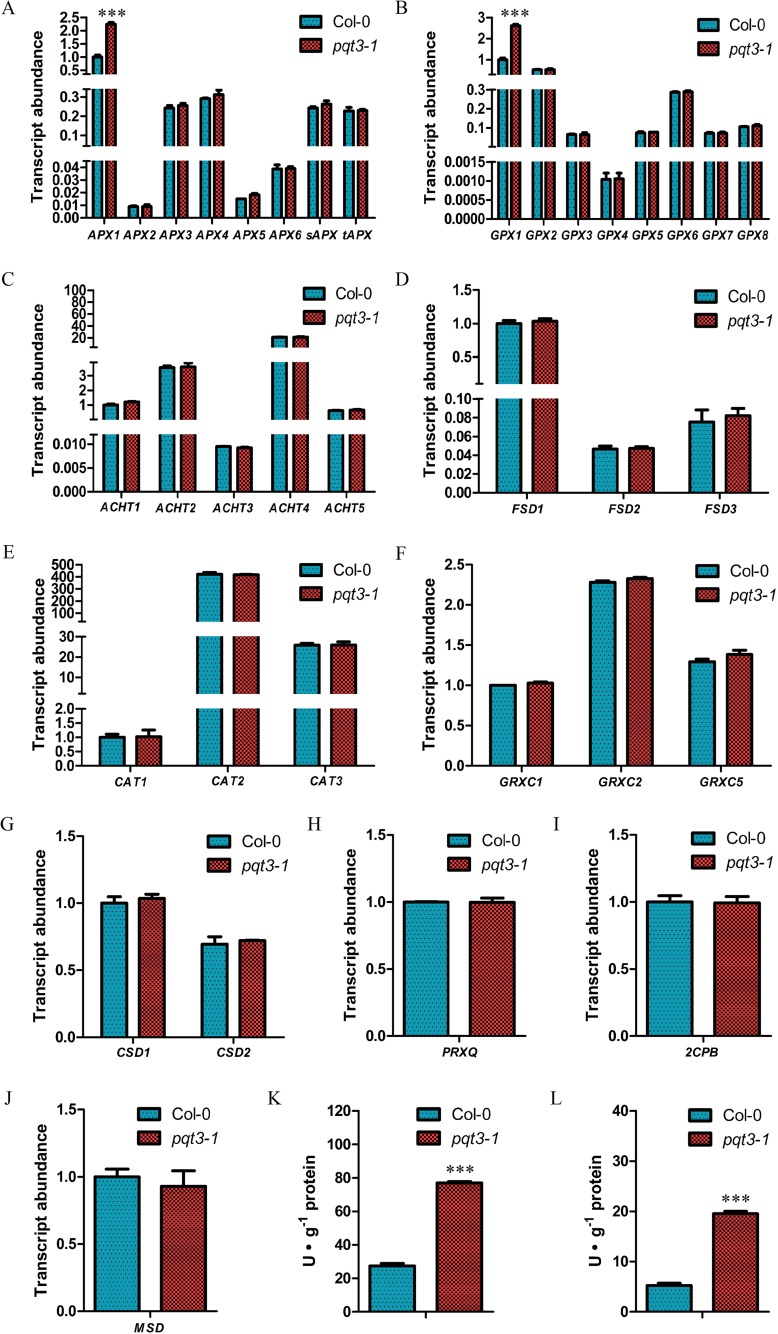
The analysis of antioxidant enzymes in wild type and *pqt3* mutant. **(A-J)** Quantitative RT-PCR analysis of transcript levels of antioxidant enzyme genes. RNA samples were isolated from 7-day-old wild type and *pqt3* seedlings for quantitative RT-PCR analysis. The transcript levels of APX **(A)**, GPX **(B)**, ACHT **(C)**, FSD **(D)**, CAT **(E)**, GRXC **(F)**, CSD **(G)**, PRXQ **(H)**, 2CPB **(I)** and MSD **(J)** were analyzed. Values are mean ± SD (n = 3 experiments, ***P < 0.001). Asterisks indicate Student’s t-test significant differences. **(K and L)** Enzyme activity of APX **(K)** and GPX **(L)** in wild type and *pqt3* mutant. The *pqt3* mutant has higher enzyme activity of APX and GPX than wild type. Values are mean ± SD (n = 3 experiments, ***P < 0.001). Asterisks indicate Student’s t-test significant differences.

Enzymatic protection systems are very important for ROS elimination. The transcript levels of ascorbate peroxidase (APX), glutathione peroxidase (GPX), catalase (CAT), cytosolic Cu/Zn SOD (CSD1), plastidic Cu/Zn SOD (CSD2), FeSOD (FSD), atypical Cys-His rich thioredoxin (ACHT), glutaredoxin C (GRXC), 2-Cys peroxiredoxin B (2CPB), peroxiredoxin Q (PRXQ) and mitochondrial MnSOD (MSD) were analyzed by quantitative RT-PCR in *pqt3* and wild type. The results show that transcript levels of *APX1* and *GPX1* were up-regulated in *pqt3* under normal conditions compared with that in the wild type (Fig 4A–4J). The elevated transcript levels of *APX1* and *GPX1* may contribute to the improved oxidative tolerance.

The enzyme activity of APX and GPX in wild type and *pqt3* mutant was also detected. The *pqt3* mutant had higher enzyme activity of APX and GPX compared with wild type (Fig 4K and 4L).

### PQT3 interacts with PRMT4b

**Fig 5:**
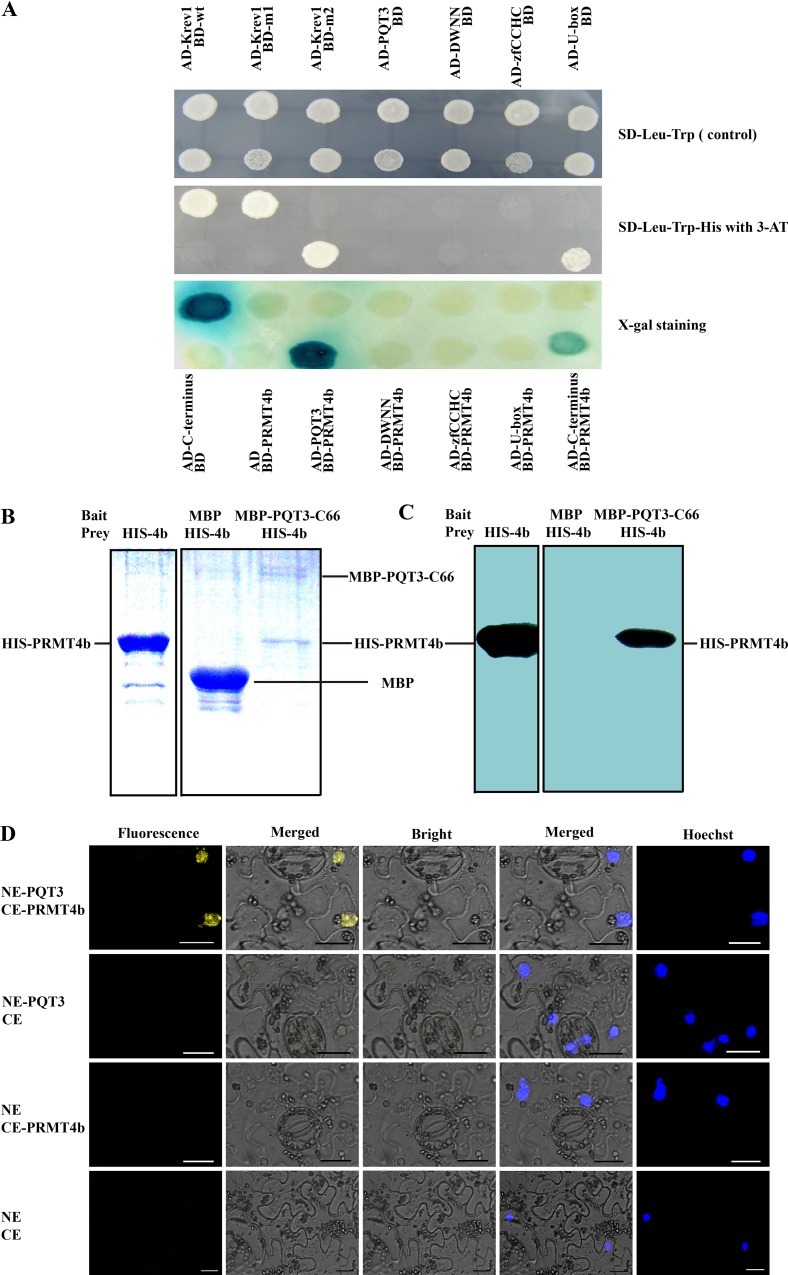
PQT3 interacts with PRMT4b. **(A)** Y2H assay. PQT3 and its four protein sections were used as the bait. PRMT4b was used as the prey. Krev1/RalGDS-wt act as strong positive control and Krev1/RalGDS-m1 act as weak positive control. Krev1/RalGDS-m2 was used for negative control. The yeast harboring various constructs was grown on SD-Leu-Trp medium (upper panel). The yeast was transferred to SD-Leu-Trp-His medium with 50 mM 3-AT (middle panel) or used for X-gal staining (lower panel). **(B and C)** The pull-down assay between PQT3 and PRMT4b. His-PRMT4b was incubated with amylose resin bound with recombinant MBP-PQT3-C66 protein. Pulled-down protein complex was detected by SDS-PAGE **(B)** and western blot using anti-His antibody **(C)**. MBP protein was used as a negative control. **(D)** BiFC assay. Different plasmid combinations were expressed in epidermal cells of *N*. *benthamiana* leaves. Yellow fluorescence protein (YFP) was observed in epidermal cell expressing both NE-PQT3 (the N-terminal part of YFP fused with PQT3) and CE-PRMT4b (the C-terminal part of YFP fused with PRMT4b). No fluorescence was observed from the negative controls: NE-PQT3 / CE, NE / CE-PRMT4b, and NE / CE. The nuclei were stained by Hoechst and the fluorescence was detected by confocal. NE indicates pSPYNE vector and CE indicates pSPYCE vector.

To study the molecular mechanisms that underlie the enhanced stress tolerance of *pqt3*, we screened cDNA library for potential candidate target proteins of PQT3 using yeast-two-hybrid (Y2H). Several proteins were isolated from the screen. Among these candidate interactors, PRMT4b, a member of arginine methyl transferase family, was frequently presented. To reveal the domain of PQT3 responsible for the interaction with PRMT4b, the PQT3 protein was divided into four parts: N-terminal DWNN, zfCCHC, U-box (RING finger), and C-terminal section containing the NLS1 and NLS2 domains, based on the predicted domains of PQT3 protein (S3A Fig). Full-length PQT3 and four protein sections were used for Y2H assay as baits. Full-length protein and the C-terminus of PQT3 were able to interact with PRMT4b in Y2H assays (Fig 5A). The interaction between PQT3 and PRMT4b was confirmed by colonies that grew on the SD-Leu-Trp-His plate with 50 mM 3-amino-1, 2, 4-triazole (3-AT) and displayed the blue color in X-gal assay (Fig 5A). However, PRMT4a was never isolated in the screening. The Y2H assay was also performed to further study the potential interaction between PQT3 and PRMT4a, since *PRMT4a* is a close related gene of *PRMT4b*. The result showed that the PQT3 did not interact with PRMT4a (S5 Fig).

The interaction between PQT3 and PRMT4b was further confirmed by protein pull-down assay *in vitro*. MBP-PQT3-C66 protein containing the NLS domain (S3A Fig) and His-PRMT4b protein were expressed in *E*. *coli* and purified subsequently. His-PRMT4b was incubated with amylose resin bound with recombinant MBP-PQT3-C66 protein. Pulled-down protein complex was detected by SDS-PAGE (Fig 5B) and western blotting using anti-His antibody (Fig 5C). The pull-down result clearly shows that PQT3 interacts with PRMT4b *in vitro*.

To determine whether the interaction also occur *in vivo*, we used the bimolecular fluorescence complementation (BiFC) system. The N-terminus of yellow fluorescent protein (YFP) was fused to full-length *PQT3* cDNA, while full-length *PRMT4b* cDNA was fused to the C-terminal region of YFP. The empty plasmids were used as negative controls. Different plasmid combinations were co-infiltrated into epidermal cell of *N*. *benthamiana* leaves. The yellow fluorescence was observed in epidermal cell contained both NE-PQT3 (the N-terminus of YFP fused with PQT3) and CE-PRMT4b (the C-terminus of YFP fused with PRMT4b) (Fig 5D). No fluorescence was observed from the negative controls (NE-PQT3/CE, NE/CE-PRMT4b and NE/CE) (Fig 5D). The nuclei were stained by Hoechst and detected by confocal. These results indicate that PQT3 can interact with PRMT4b in the nucleus of plant cell.

### PQT3 has E3 ubiquitin ligase activity and can ubiquitinate PRMT4b

**Fig 6:**
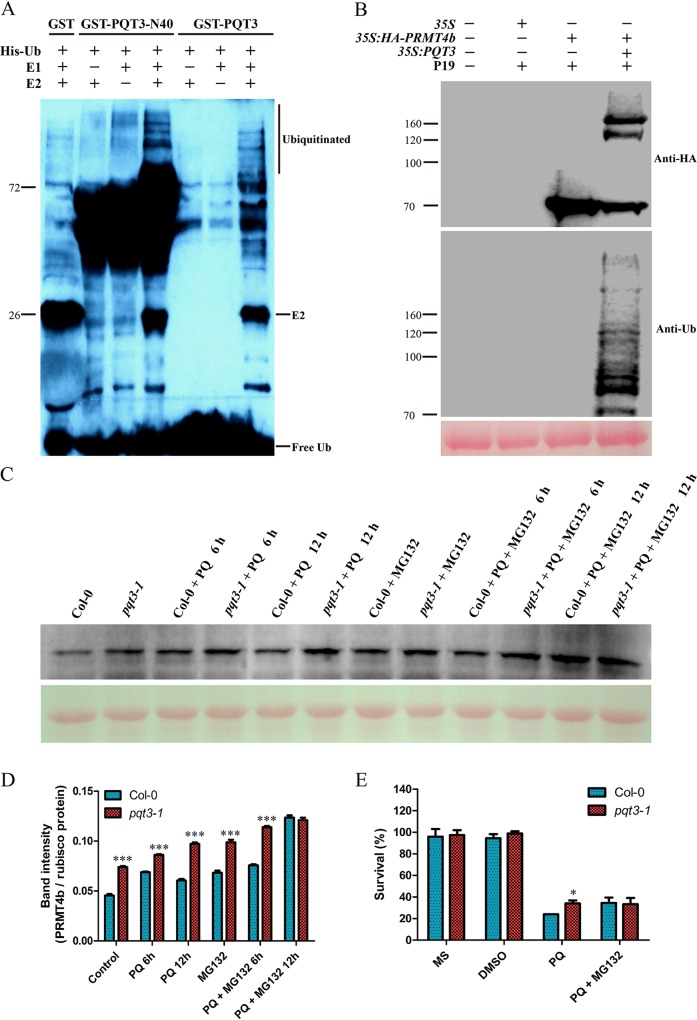
PRMT4b is a substrate recognized by PQT3. **(A)** *In vitro* ubiquitination assay. GST-PQT3-N40 and GST-PQT3 were expressed in *E*. *coli* and purified. Nickel-HRP was used to detect His-tagged ubiquitin. The ubiquitination activity of PQT3 was observed only in the presence of E1 (from wheat), E2 (UBCh5b, from human), and 6×His-tagged ubiquitin (Ub). GST was used as negative control. The numbers on the left indicate the molecular masses of marker proteins. **(B)** *In planta* ubiquitination assay. Total protein samples were isolated from the infiltrated parts of *N*. *benthamiana* leaves 1 day after agroinfiltration. The total protein was analyzed via western blot using anti-HA antibody (upper panel). Immunoprecipitated samples were analyzed using western blot with anti-ubiquitin antibody subsequently (middle panel). Ponceau S staining of the Rubisco protein was shown as a loading control (lower panel). The numbers on the left indicate the molecular masses of marker proteins. **(C and D)** The effect of proteasome inhibitor MG132 on the protein level of PRMT4b in wild type and *pqt3-1* mutant without or with paraquat treatment in time-course. Wild type and *pqt3-1* mutant were grown for 14 days, then the seedlings were treated without or with 6 μM paraquat for 6 or 12 h in presence or absence of 50 μM proteasome inhibitor MG132. The total proteins were extracted. Western blot was performed using anti-PRMT4b antibody **(C)**. Ponceau S staining of the rubisco protein serves as a loading control. Band densities were quantified using Quantity One software (Bio-Rad, USA) **(D)**. Values are mean ± SD (n = 3 experiments, ***P < 0.001). Asterisks indicate Student’s t-test significant differences. **(E)** The survival ratio of wild type and *pqt3* mutant germinated and grown on MS medium containing 0 μM or 1 μM paraquat without or with 15 μM MG132 for 7 days were counted. DMSO was used as control. Values are mean ± SD (n = 30 plants, *P < 0.05). Asterisk indicates Student’s t-test significant difference.

Not all the proteins with the predicted RING domain function as an ubiquitin ligase [62]. The E3 activity of PQT3 was determined via self-ubiquitination system. Both full-length (GST-PQT3) and C-terminal deletion (GST-PQT3-N40) proteins showed the E3 ubiquitin ligase activity (Fig 6A and S3B Fig). The ubiquitinated bands of PQT3 were detected by western blotting in the presence of E1 (from wheat), E2 (UBCh5b, from human), and 6×His-tagged ubiquitin (UBQ14, from Arabidopsis). When any of essential reaction components was missing, self-ubiquitination of PQT3 was not detected (Fig 6A).

To determine whether PRMT4b is a substrate recognized by PQT3, we resorted to the *in planta* ubiquitination assay [63]. Leaf infiltration was conducted via *Agrobacterium tumefaciens* strains containing different combination of constructs. The infiltrated parts of *N*. *benthamiana* leaves were harvested. Total protein was extracted and detected via western blotting with anti-HA antibody. A smear of bands, which were larger than the size of HA-PRMT4b and showed the features of ubiquitinated form of the PRMT4b proteins, could be detected by anti-HA antibody in the samples co-infiltrated with PQT3 and HA-PRMT4b (Fig 6B). The cell lysates were immunoprecipitated with anti-HA antibody subsequently. Immunoprecipitated samples were detected via western blotting with anti-ubiquitin antibody. In the PQT3-PRMT4b co-infiltration sample, these high molecular size bands could also be detected by anti-ubiquitin antibody (Fig 6B). These results indicated that these high molecular size bands were ubiquitinated forms of PRMT4b. PQT3 protein could ubiquitinate the PRMT4b protein in tobacco. The decline of PRMT4b protein was also found in the samples co-infiltrated with PQT3 and HA-PRMT4b (Fig 6B).

In addition, PRMT4b protein levels in different *PQT3* genetic background under MG132 treatments were also supportive of PRMT4b as a substrate of PQT3. As shown in the Fig 6C and 6D, the protein level of PRMT4b in *pqt3-1* was higher than that of the wild type under normal conditions. Under paraquat treatment, it remained lower in wild type than that of *pqt3-1* at the same time point, although protein level of PRMT4b increased gradually in wild type and *pqt3-1* with the prolonged paraquat treatment (Fig 6C and 6D). The transcript level of *PRMT4b* was unlikely to cause the observed difference of protein levels between the mutant and wild type (S6 Fig). Under MG132 treatment, the protein level of PRMT4b was increased in both wild type and *pqt3-1* mutant. When the seedlings were co-treated with paraquat and MG132 for 12 h, PRMT4b was accumulated in both wild type and *pqt3-1* mutant, and no significant difference of PRMT4b protein level was found between the wild type and *pqt3-1* mutant (Fig 6C and 6D) because the degradation of the PRMT4b protein through ubiquitination-26S proteasome pathway was inhibited by MG132.

In order to confirm further the PQT3-dependent ubiquitination of PRMT4b, the phenotype of wild type and *pqt3-1* mutant treated without or with paraquat in presence or absence of proteasome inhibitor MG132 was studied. Survival ratio of *pqt3-1* seedlings was higher than that of wild type under paraquat treatment. No significant difference of survival ratio could be observed when the wild type and *pqt3-1* seedlings were co-treated with paraquat and MG132 (Fig 6E). The wild type gained enhanced paraquat tolerance under MG132 treatment to the level of *pqt3-1* mutant.

### *PRMT4b* is a positive regulator in the oxidative stress tolerance

**Fig 7:**
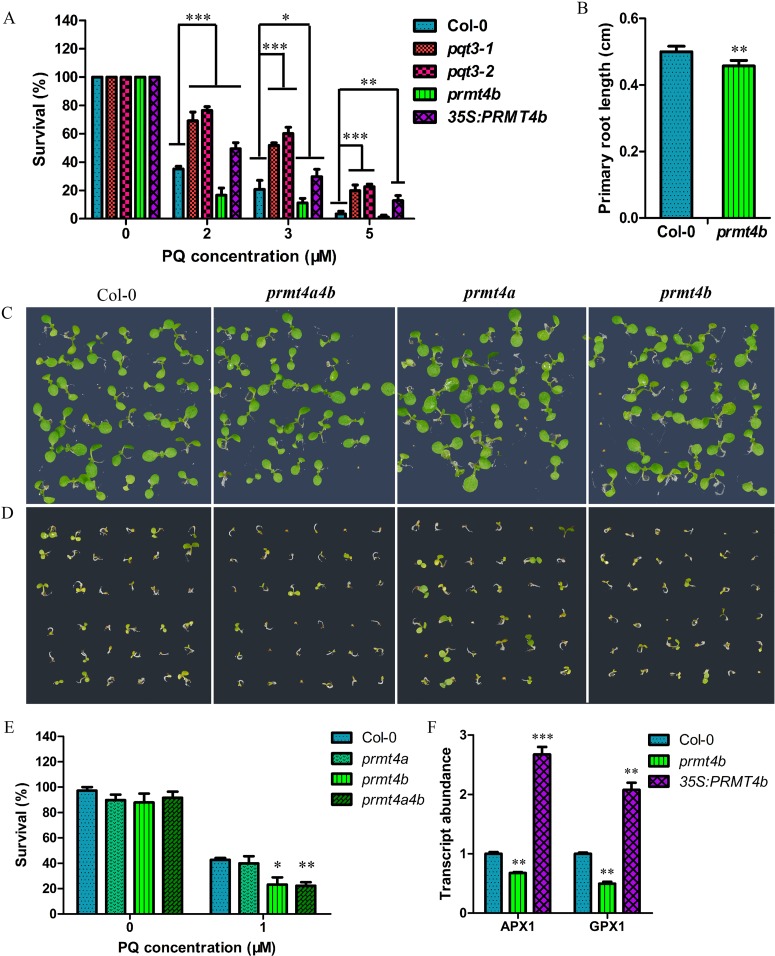
PRMT4b is involved in oxidative tolerance of Arabidopsis. **(A)** Paraquat tolerance assay. Survival ratio of wild type, *pqt3-1* mutant, *pqt3-2* mutant (Salk_065409), *prmt4b* mutant and *35Spro*:*PRMT4b* grown on 0, 2, 3, or 5 μM paraquat medium were counted. The assay was repeated for three times. Values are mean ± SD (n = 50 plants, *P < 0.05, **P < 0.01, ***P < 0.001). Asterisks indicate Student’s t-test significant differences. **(B)** Primary root elongation of wild type and *prmt4b* mutant seedlings grown on MS without or with 150 μM CdCl_2_ was measured. Values are mean ± SD (n = 30 plants, **P < 0.01). Asterisks indicate Student’s t-test significant differences. **(C-E)** The phenotype of wild type, *prmt4a*, *prmt4b*, and *prmt4aprmt4b* grown on MS without **(C)** or with 1 μM paraquat **(D)**. The survival ratio of wild type, *prmt4a*, *prmt4b*, and *prmt4aprmt4b* germinated and grown on MS medium containing 0 μM or 1 μM paraquat for 5 days were counted **(E)**. Values are mean ± SD (n = 36 plants, *P < 0.05, **P < 0.01). Asterisks indicate Student’s t-test significant differences.**(F)** The transcript levels of *APX1* and *GPX1* in wild type, *prmt4b* mutant and *35Spro*:*PRMT4b*. RNA samples were isolated from 7-day-old seedlings for quantitative RT-PCR analysis. Values are mean ± SD (n = 3 experiments, **P < 0.01, ***P < 0.001). Asterisks indicate Student’s t-test significant differences.

To reveal whether PRMT4b could play any roles in oxidative tolerance of plants, we obtained the *prmt4b* mutant and *35Spro*:*PRMT4b* lines (S1G, S1H and S1L Fig) and observed the phenotypes of *prmt4b* and *35Spro*:*PRMT4b* under different concentrations of paraquat treatment. Survival ratio of wild type, *pqt3-1*, *pqt3-2*, *prmt4b* and *35Spro*:*PRMT4b* were counted (Fig 7A). *35Spro*:*PRMT4b* had similar phenotype as *pqt3-1* and *pqt3-2*, while the *prmt4b* mutant was more sensitive to paraquat treatment than wild type (Fig 7A). Under CdCl_2_ treatment, primary root elongation of *prmt4b* mutant was also slower than that of wild type (Fig 7B). Furthermore, the overexpression lines of *PRMT4b* were analyzed under other stress conditions. *35Spro*:*PRMT4b* increased tolerance to CdCl_2_ and NaCl stresses compared with wild type. The *35Spro*:*PRMT4b* showed the opposite phenotype of the *35Spro*:*PQT3* which was more sensitive to CdCl_2_ and NaCl stresses as compared with wild type (S7 Fig). These results show that PRMT4b is a positive regulator for plant oxidative tolerance, which is also consistent with the function of PQT3. The *prmt4a* mutant and *prmt4aprmt4b* double mutants were subsequently examined (S1I–S1K Fig). The *prmt4aprmt4b* double mutants had the similar phenotype to *prmt4b* under paraquat treatment, and the knockout of *PRMT4a* did not affect the oxidative tolerance (Fig 7C–7E). In addition, the transcript levels of *APX1*, *GPX1* and other antioxidant enzyme genes were down-regulated in *prmt4b* mutant and up-regulated in *35Spro*:*PRMT4b* as compared with that in the wild type (Fig 7F and S8 Fig).

### Increased H3R17 methylation on *APX1* and *GPX1* chromatin in *pqt3*

**Fig 8:**
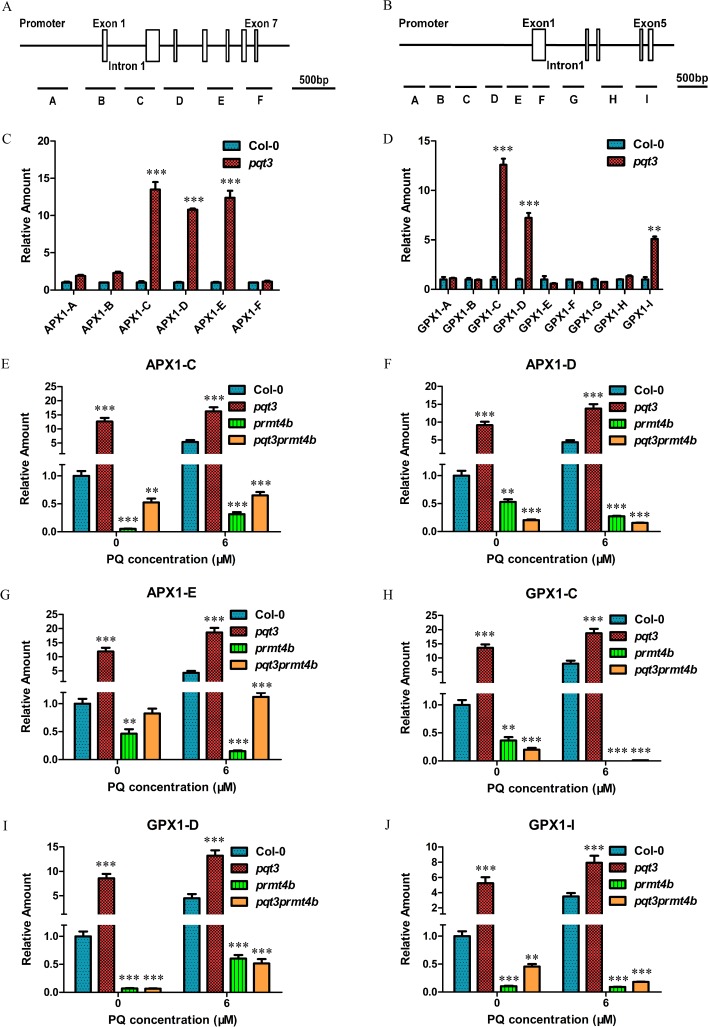
Arg-17 methylation in specific regions of *APX1* and *GPX1* chromatin is increased in *pqt3* mutant. **(A and B)** The illustration of *APX1* and *GPX1* chromatin. A to F represent different regions of *APX1* chromatin **(A)**. A to I represent different regions of *GPX1* chromatin **(B)**. Promoters (black lines), exons (white boxes), and introns (black lines) of *APX1* and *GPX1* were shown. **(C and D)** ChIP-PCR assay. Quantitative PCR was performed to verify each chromatin region of *APX1* and *GPX1* using specific primers. Fragments C, D and E in *APX1* chromatin were enriched by anti-H3R17 antibodies in *pqt3* mutant **(C)**. Fragments C, D and I in *GPX1* chromatin were enriched by anti-H3R17 antibodies in *pqt3* mutant **(D)**. *UBQ5* was used as an internal control. Values are mean ± SD (n = 3 experiments, **P < 0.01, ***P < 0.001). Asterisks indicate Student’s t-test significant differences.**(E-G)** The enrichment of C fragment **(E)**, D fragment **(F)** and E fragment **(G)** in *APX1* chromatin was analyzed in wild type, *pqt3* mutant, *prmt4b* mutant and *pqt3prmt4b* double mutants without or with paraquat treatment. *UBQ5* was used as an internal control. Values are mean ± SD (n = 3 experiments, **P < 0.01, ***P < 0.001). Asterisks indicate Student’s t-test significant differences. **(H-J)** The enrichment of C fragment **(H)**, D fragment **(I)** and I fragment **(J)** in *GPX1* chromatin was analyzed in wild type, *pqt3* mutant, *prmt4b* mutant and *pqt3prmt4b* double mutants without or with paraquat treatment. *UBQ5* was used as an internal control. Values are mean ± SD (n = 3 experiments, **P < 0.01, ***P < 0.001). Asterisks indicate Student’s t-test significant differences.

The transcript level of *APX1* and *GPX1* was higher in *pqt3* than that in wild type under normal conditions (Fig 4A and 4B), which could be regulated by PRMT4b. The modification status of H3R17me2a in the chromatin of *APX1* and *GPX1* was compared between wild type and *pqt3* mutant via chromatin immunoprecipitation (ChIP) assays. ChIP assays were performed with wild type and *pqt3* plants using antibody against H3R17me2a. As shown in the Fig 8A and 8B, the *APX1* and *GPX1* chromatin was divided into different regions and the enriched chromosome fragments were detected by quantitative RT-PCR. The results showed that histone H3R17me2a modification of *APX1* and *GPX1* chromatin was increased in *pqt3* mutant (Fig 8C and 8D). The enriched chromosome fragments of *APX1* (C, D and E fragments) and *GPX1* (C, D and I fragments) were further analyzed in *prmt4b* mutant and *pqt3prmt4b* double mutants without or with paraquat treatment (Fig 8E–8J). Histone H3R17me2a modification of *APX1* (C, D and E fragments) and *GPX1* (C, D and I fragments) chromatin was decreased in *prmt4b* mutant and *pqt3prmt4b* double mutants without paraquat treatment as compared with wild type. It remained lower in wild type than that of *pqt3* mutant, although H3R17me2a modification of *APX1* (C, D and E fragments) and *GPX1* chromatin (C, D and I fragments) in both wild type and *pqt3* mutant were enhanced under paraquat treatment. H3R17me2a modification of *APX1* (C, D and E fragments) and *GPX1* chromatin (C, D and I fragments) was kept at a low level, although the modification in *prmt4b* mutant and *pqt3prmt4b* double mutants could also be affected by paraquat treatment. These results suggest that PRMT4b may target *APX1* and *GPX1* to enhance the oxidative tolerance by increasing asymmetric dimethylation of H3 at R-17 in *APX1* and *GPX1* chromatin.

### *pqt3prmt4b* double mutants in oxidative tolerance

**Fig 9:**
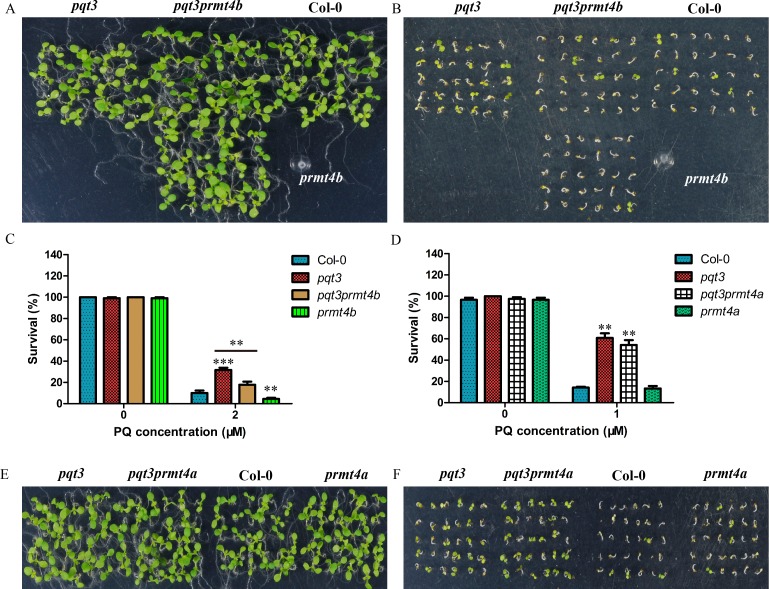
The phenotypes of *pqt3prmt4b* and *pqt3prmt4a* double mutants under paraquat treatment. **(A and B)** The phenotypes of *pqt3*, *pqt3prmt4b*, wild type and *prmt4b* seedlings grown on MS without **(A)** or with 2 μM paraquat **(B)**. **(C)** The survival ratio of *pqt3*, *pqt3prmt4b*, wild type and *prmt4b* under 2 μM paraquat treatment for 7 days were counted. Values are mean ± SD (n = 30 plants, **P < 0.01, ***P < 0.001). Asterisks indicate Student’s t-test significant differences. **(D)** The survival ratio of *pqt3*, *pqt3prmt4a*, wild type and *prmt4a* under 1 μM paraquat treatment for 7 days were counted. Values are mean ± SD (n = 30 plants, **P < 0.01). **(E and F)** The phenotypes of *pqt3*, *pqt3prmt4a*, wild type and *prmt4a* seedlings grown on MS without **(E)** or with 1 μM paraquat **(F)**.

To confirm further that PRMT4b is the target of PQT3, the *pqt3prmt4b* double mutants were obtained (S1M and S1N Fig). The survival ratio of *pqt3prmt4b* under paraquat treatment was intermediate between *pqt3* and *prmt4b*, which demonstrates that the PRMT4b protein is one of the targets of PQT3 and suggests that PQT3 may also target other proteins that contribute to the tolerance to oxidative stress (Fig 9A–9C).

We also obtained the *pqt3prmt4a* double mutants (S1O and S1P Fig) and found that the survival ratio of *pqt3prmt4a* under paraquat treatment had no significant difference from that of *pqt3* mutant (Fig 9D–9F), again indicating that *PRMT4a* is not involved in oxidative stress response.

## Discussion

PQT3 is a member of Arabidopsis RING-finger/U-box E3 ligase family. Secondary structure prediction using InterProScan protein sequence analysis software revealed four conserved domains including DWNN (domain with no name), zinc finger domain, RING-finger domain, and U-box domain in N-terminus of PQT3 protein (S9A and S9B Fig). DWNN is a novel ubiquitin-like domain, which is a highly-conserved domain in eukaryotic plants and animals [64]. The DWNN domain of PQT3 contains 76 amine acids. DWNN domain is only found in the N-terminus of the members in the splicing-associated RBBP6 (Retinoblastoma Binding Protein 6) protein family [64]. The RING-finger domain was also found in RBBP6 protein family. The existence of the RING-finger domain suggests that DWNN domain may play its role as ubiquitin-like regulatory factors [64]. CCHC-type Zinc finger domain is also known as zinc knuckle, which can be found in a large number of RNA binding proteins [65, 66]. As mentioned above, RING-finger domain can combine with the E2 in the cascade reaction of ubiquitination system,while U-box is a modified RING-finger [67]. In addition, two predicted NLS sequences (471–477 and 696–711 amino acids) were also found in the C-terminus of PQT3, which is consistent with nuclear localization of the protein (Fig 2H–2P and S9A and S9B Fig). Phylogenetic tree analysis further revealed the high homology proteins of PQT3 in other species (S9C Fig). DWNN domain and RING-finger/U-box domain in N-terminus of PQT3 were highly conserved in homologous proteins in different plant species (S10 Fig). The PQT3 may play its role as an ubiquitin ligase in different species and its function may be conserved throughout the plant kingdom.

*In vitro* ubiquitination assay shows that PQT3 has the E3 ligase activity (Fig 6A). We noticed that the *in vitro* activity was not as high as expected, which could be contributed by suboptimal reaction conditions such as E2 source. This E3 activity was confirmed by *in planta* ubiquitination assay, in which PRMT4b protein could be ubiquitinated by expressed PQT3 protein in *N*. *benthamiana* (Fig 6B). Consistent with this result, *pqt3-1* mutant had higher protein level of PRMT4b than the wild type, as the PRMT4b protein was degraded by PQT3 in wild type under normal conditions (Fig 6C and 6D). The transcript level of *PRMT4b* could be induced in wild type under paraquat treatment (S6 Fig), while the transcript level of *PQT3* was decreased by paraquat treatment (Fig 3A). In this case, PQT3-mediated PRMT4b degradation could be weakened in wild type. Consequently, the level of PRMT4b protein in wild type was elevated, but lower than that of *pqt3* (Fig 6C and 6D). The proteasome inhibitor MG132 could block the degradation of the PRMT4b protein in wild type and enhance paraquat tolerance of wild type (Fig 6C–6E). Taken together, PQT3, as an E3 ubiquitin ligase, play its role in oxidative tolerance through the ubiquitination-degradation of PRMT4b.

A series of environmental stress could lead to oxidative damage to plants [10, 13, 68]. Both biotic stress and abiotic stress result in the production of ROS which in excess cause oxidative stress. Besides paraquat tolerance, *pqt3* mutants have enhanced tolerance to various environment stresses (Fig 1 and S2 Fig). Further studies may reveal new mechanisms of PQT3 in multiple stress tolerance. Analysis of PQT3-interacting proteins may be a good start point to further understand the function of *PQT3*. In the screen for PQT3-interacting proteins with Y2H, we isolated 66 positive colonies based on the expression of reporter genes included His3 and LacZ. Several proteins were identified from these positive colonies repeatedly. The most frequent interacting partner is 20S core protease subunit of 26S proteasome. The result suggested that PQT3 act as an E3 ubiquitin ligase. Among these proteins, PRMT4b, as previously mentioned, may be responsible for the increased degree of Arg-17 methylation which may further regulate *APX1* and *GPX1* genes to enhance oxidative tolerance of plants (Figs 4A, 4B and 8). It has been reported that arginine methylation was involved in signal transduction, transcriptional control, DNA repair, RNA processing, and nuclear transport [17, 19, 23, 69–72]. The functions of PRMTs have been extensively analyzed [15, 19, 20]. Here, the *prmt4b* mutant was found to be more sensitive to paraquat and CdCl_2_ treatment than wild type (Fig 7A–7E). Thus, PRMT4b plays a role in oxidative stress response of plants. It is known that PRMT4-mediated methylation at Arg-17 of histone H3 is linked to transcription activation [73]. The Arg-17 of histone H3 is the major site of PRMT4-mediated methylation, although it was reported that other protein site could also be methylated by PRMT4 [74–76]. We suggest that the increased Arg methylation degree of specific regions in *APX1* and *GPX1* chromatin was caused by PRMT4b (Fig 8).

The increased transcript level of *APX1* and *GPX1* may be caused by PRMT4b-mediated histone Arg methylation (Figs 4A, 4B and 8). In presence of ascorbate as electron donor, the cytosolic enzyme, APX1, catalyze the degradation of H_2_O_2_ [77]. The response of *APX1* to oxidative stress has been studies in Arabidopsis. The crucial role of APX1 in multiple stress response was reported [78]. It could be activated by multiple stresses to protect plants against oxidative stress [79–81]. The tobacco overexpressing *APX1* could be more tolerant against UV-C-caused oxidative damage [82]. GPXs also have important functions in oxidative signaling, which can protect plants from harmful effects of excessive oxidation [83]. It has also been reported that enhanced peroxide scavenging and decreased oxidative damage was found in transgenic tobacco seedlings overexpressing tobacco glutathione S-transferase that showed glutathione peroxidase (GPX) activity [84].

The *pqt3* mutants also have late-flowering phenotype (Fig 1D). It has been reported that *prmt4aprmt4b* double mutants display late-flowering phenotype [15]. The Y2H result showed that the PQT3 can not interact with PRMT4a (S5 Fig). As compared with wild type, the *prmt4a* mutant also has no significant difference in the oxidative tolerance (Fig 7C–7E). The phenotype of *pqt3prmt4a* double mutants demonstrate that PRMT4a protein was not involved in the response regulation of oxidative stress by PQT3 (Fig 9D–9F). The late-flowering phenotype of *pqt3* may be regulated by other mechanisms, rather than *PRMT4a* and *PRMT4b*. The flowering-related transcription factor AGAMOUS (AG) was found to be a potential target interacted with PQT3 in Y2H library screening. AG is involved in carpel development, leaf development, identification of floral organs, and stamen development [85]. Targeted removal of AG by PQT3 may be related to the late-flowering phenotype of *pqt3*. In addition, one member of a large G protein family and the proteins with unknown function were also found to be potential targets interacted with PQT3 in Y2H library screening. By interacting with different partners, PQT3 may mediate multiple functions in diverse biological processes.

The *pqt3* has enhanced tolerance to multiple stresses. PQT3 is also down-regulated by various oxidative stresses. PQT3 act as negative regulator in multiple stress responses. The interacting partner will be further analyzed to reveal the molecular function of *PQT3*. As research continues, other combinations, also targeted by PQT3, may be revealed, where PQT3 act as a positive regulator. We could improve crop tolerance to multiple stresses through *PQT3* mutation. More candidate genes could be further determined from the potential targets interacted with PQT3. These genes may also have high application value after the revelation of molecular mechanisms.

**Fig 10:**
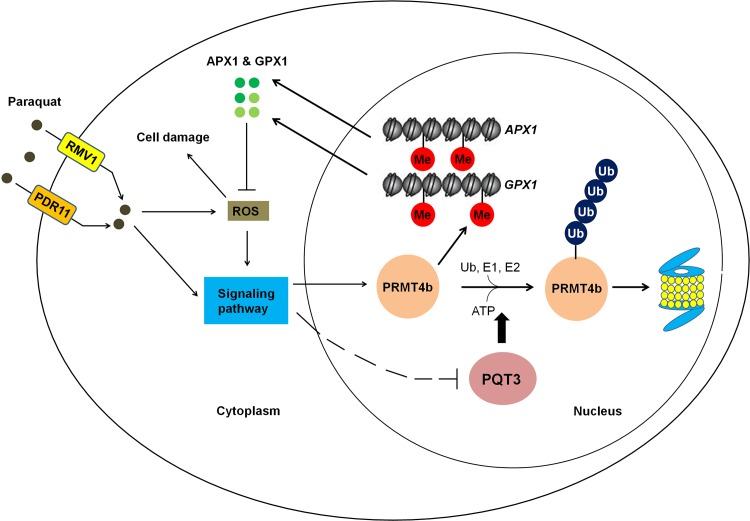
A working model for PQT3 acting as a negative regulator of oxidative stress response. Many environmental stresses cause oxidative stress in plants. Under oxidative stress, stress signaling up-regulates *PRMT4b* expression and down-regulates *PQT3* expression, leading to higher PRMT4b activity that will activate *APX1* and *GPX1* and enhance antioxidation capacity of APX1 and GPX1. When oxidative stress is diminished, *PRMT4b* expression is decreased and *PQT3* expression is increased. As a result, PQT3 activity is increased, leading to faster removal of PRMT4b via 26S proteasome. Together with decreased *PRMT4b* expression, PRMT4b activity will rapidly drop, leading to decreased expression of *APX1* and *GPX1*. The activated response of oxidative stress is then switched off.

In conclusion, paraquat may enter the cell through plasma membrane-localized paraquat transporters PDR11 and RMV1 [59, 86]. The intracellular paraquat may further activate different downstream signaling pathways to regulate the expression of *PQT3* and *PRMT4b*. In addition, paraquat stress increases the generation of ROS. ROS is also an important signal molecule that mediate the responses to environmental stress [87]. ROS-activated signaling pathways may also be responsible for the regulation of *PQT3* and *PRMT4b*. The oxidative stress activates the expression of *PRMT4b*, represses the expression of *PQT3*, and weakens PQT3-mediated the ubiquitinated degradation of PRMT4b, synergistically resulting in increased accumulation of PRMT4b. Consequently, the increased level of PRMT4b protein may lead to higher degree of histone methylation on the *APX1* and *GPX1* chromatin. As a result, the transcription of *APX1* and *GPX1* is activated, leading to more APX1 and GPX1 enhancing oxidative tolerance of plants. When the stress disappears, transcription repression of *PQT3* by oxidative stress is removed. The function of PQT3, as a negative regulator of oxidative stress response, is restored. The PRMT4b is then degraded by PQT3 in ubiquitination pathway. The activated response to oxidative stress is switched off. We propose a working model for PQT3 as a negative regulator of oxidative stress response (Fig 10).

## Materials and Methods

### Mutant screen from the activation-tagging library

Approximately 55,000 individual lines were screened for paraquat tolerant mutant, which came from the activation-tagging library constructed with Arabidopsis Columbia ecotype and pSKI015 vector [59].

### Plant material and growth conditions

All Arabidopsis transgenic lines and mutants were based on the genetic background of Columbia ecotype used as wild type in the study. Salk_065409 (*pqt3-2*), Salk_097442C (*prmt4b*) and Salk_033423 (*prmt4a*) were obtained from ABRC (Arabidopsis Biological Resource Center). The seeds were sterilized in 10% bleach for 10 min. Then the seeds were washed for 5 times at least with sterile water. For vernalization, the seeds were kept in the dark with water at 4°Cfor 3 days to ensure the synchronous germination. Sterile seeds were germinated on MS medium. The seedlings were grown at 22°C under 16-h-light /8-h-dark cycle with light intensity 100 μE m^-2^ s^-1^.

### Transformation of Arabidopsis

The constructs were electroporated into competent cell of *Agrobacterium tumefaciens* C58C1. The floral-dip method was used to transfer these constructs into Arabidopsis as described [88, 89].

### PCR analysis

The different tissues of plants were used for RNA extraction with Trizol method. Then RNA reverse reaction was carried out by TransScript Kit (TransGen Biotech). Specific primers were designed for RT-PCR analysis and the PCR products were detected by agarose gel electrophoresis. Applied Biosystem Step One real-time PCR system was used for the quantitative RT-PCR detection with specific primers listed in the S1 Table and Premix Ex Taq II SYBR (TaKaRa). *UBQ5* was used as the internal control.

### Identification of mutants, FC line, *35Spro*:*PQT3*, and *35Spro*:*PRMT4b*.

The identification of homozygous mutants was performed by genomic PCR for T-DNA insertion lines: Salk_065409, Salk_097442C and Salk_033423 [90]. RT-PCR was carried out as previously described to confirm the results of genomic PCR. The *35Spro*:*PQT3* and *35Spro*:*PRMT4b* plasmids were transformed into Col-0 to obtain the overexpression lines of *PQT3* and *PRMT4b*. For the FC line, the *35Spro*: *PQT3* was transformed into *pqt3* mutant, and the line with the same expression level of *PQT3* as wild type was chosen and used as FC line. *35Spro*: *PQT3*, *35Spro*: *PRMT4b* and FC line were identified by glufosinate screening and quantified by RT-PCR or quantitative RT-PCR.

### Stress tolerance assay

The seeds were germinated on MS medium containing different concentrations of paraquat, mannitol, CdCl_2_ and NaCl, respectively. The phenotype was observed and survival ratio was scored at the indicated time points.

For drought tolerance assay, the wild type, *pqt3-1* mutant and *pqt3-2* mutant seeds were germinated in one pot at same density. The 15-day-old seedlings were used for drought tolerance assay. Watering was withheld for another 15 days before re-watering. The photos were taken before re-watering and after re-watering for 1 day and 7 days. The survival ratio was scored after re-watering for 1 day and 7 days.

### DAB staining

DAB staining was performed as described [91]. DAB staining solution (pH 6.0) was prepared by adding 0.05% (v/v) Tween-20 and 10 mM Na_2_HPO_4_ to the DAB solution (1 mg/ml DAB, pH3.0). For each treatment condition, at least 3 leaves per plant were obtained from 3 independent plants for each line (Col-0, *pqt3-1* and *pqt3-2*). Arabidopsis leaves from different lines were treated using MS liquid medium without or with 6 μM paraquat for 12 hr or 24 hr. These leaves were stained in 6-well culture plates with DAB staining solution subsequently and 10 mM Na_2_HPO_4_ (pH 6.0) was used as the negative control. The 6-well plates were covered with aluminum foil and placed on a shaker for 4–5 h. Follow the incubation, the bleaching solution (glycerol: acetic acid: ethanol = 1:1:3) was used in the discoloration after DAB staining solution was removed. The 6-well plates were placed into boiling water bath at 95°C for 15–20 min. Discoloration process was repeated using fresh bleaching solution. The brown precipitate caused by the reaction between DAB and H_2_O_2_ could be observed on the leaves. Photos were taken using a camera. Special attention should be paid to light avoidance through the whole operation. The experiment was repeated for three times.

### The detection of GUS activity

The promoter of *PQT3* was cloned into pCB308R [92, 93]. The transgenic lines containing *PQT3pro*: *GUS* were isolated by glufosinate screening. The T2 population was used for GUS staining. Histochemical staining for GUS activity in Arabidopsis was carried out as described previously [94] and GUS staining solution was prepared as described before [59]. The experimental materials were incubated in staining solution at 37°C. Then Arabidopsis tissues were destained and stored in 70% ethanol. The GUS activities of individual parts were observed via a light microscope Axio skop2 plus (ZEISS, Germany) with a video camera.

### Subcellular localization assay

The full length CDS of *PQT3* was cloned into the binary vector pCB2008E to construct the PQT3-GFP fusion vector [93]. After the detection of gene sequencing for inserted sequence, the recombinant plasmid was transducted into the epidermal cells of onion along with particle gun bombardment for transient expression assay. PQT3-GFP was also transferred into Col-0 via floral-dip method to create transgenic plant for the analysis of PQT3-GFP fusion protein localization. ZEISS fluorescence microscope (Axio skop2 plus) with video camera was used to observe the green fluorescence in both the epidermal cells of onion and the root cells of Arabidopsis transgenic lines.

### Enzyme activity assay

For APX and GPX enzyme activity assay, Arabidopsis seedlings were ground in liquid nitrogen and resuspended in precooling Enzyme extraction buffer (50 mM phosphate buffer (Na_2_HPO_4_-NaH_2_PO_4_), pH = 7.0; 2% (w/v) polyvinylpolypyrrolidone (PVPP); 0.05% (v/v) TritonX-100; 1 mM EDTA and 1 mM ascorbic acid) on ice. Extraction solution was centrifuged for 20 min (16,000 g/min, 4°C). The protein concentration of supernatant was detected by One-drop micro-ultraviolet spectrophotometer and SDS-PAGE before the supernatant was used for enzyme activity analysis. The detection of APX activity was performed as described with modifications [95]. For APX activity, 50 μl enzyme and 2950 μl reaction mixture (50 mM Tris-HCl, pH7.0; 0.1 mM EDTA; 0.1 mM H_2_O_2_ and 0.5 mM ascorbic acid) was mixed. The decreased OD_290_ was recorded per 10 s. The enzyme amount oxidized 1 mM AsA in one minute set as one activity unit (U) of APX. The APX activity was defined as U • g^-1^ protein. GPX activity was measured indirectly through the detection of glutathione reductase (GR) activity using GPX Activity Measurement Kit (Beyotime Biotech, China). GR activity was detected as described with modifications [96]. The OD_340_ was recorded per 30 s. The enzyme amount consumed 1 mM NADPH in one minute set as one activity unit (U) of GPX. The GPX activity was also defined as U • g^-1^ protein.

### Western blot

For western blot analysis, proteins were electroblotted from 12% acrylamide gel to nitrocellulose membrane (Immobilon-P, MILLIPORE Corporation, USA) after the separation of SDS-PAGE. Antibodies used in western blot were as follows: anti-HA antibody (M20003, Mouse mAb, Abmart, Shanghai, China), 1:1,000 for western blot; anti-PRMT4b antibody, 1:500 for western blot; anti-Ubiquitin antibody (ab7254, abcam, USA), 1:1,000 for western blot; anti-His antibody (M30111, Mouse mAb, Abmart, Shanghai, China), 1:1,000 for western blot and goat anti-mouse lgG-HRP (Santa Cruz Biotechnology, USA), 1:5,000 for western blot. Image Quant LAS 4000 (GE, USA), as the CCD camera system, was used for the result examination with Super Signal West Femto Trial Kit (Thermo, USA). Band densities were quantified via Quantity One software (Bio-Rad, USA).

### *In vitro* E3 ubiquitin ligase activity assay

The *in vitro* E3 ubiquitin ligase activity assay was carried out as described previously [43]. GST-PQT3 fusion protein was obtained from *E*. *coil* and purified subsequently. His-tagged ubiquitin of Arabidopsis (UBQ14) was also expressed using bacterial expression system and purified. In addition, the wheat (*Triticum aestivum*) E1 (GI: 136632) and human E2 (UBCh5b) were also used in the reaction. Reactions were performed for 1.5 h at 30°C. For the immunoblot, His-tagged ubiquitin of Arabidopsis was detected using Nickel-HRP (Kirkegaard & Perry Laboratories, nickel–nitrilotriacetic acid agarose conjugated to horseradish peroxidase).

### Y2H screening and confirmation

The bait plasmid was transformed into Mav203 strain of yeast, which was constructed with pDEST32 vector and the full-length *PQT3* cDNA. Yeast two-hybrid screening was performed using two-hybrid cDNA library of Arabidopsis. The cDNA library containing fragments of fusion proteins composed of prey proteins and GAL4—AD was used to transform Mav203 cells harboring bait plasmid in Y2H assay. Positive clones were screened using SD/-Leu-Trp-His and X-gal assay. Then it was identified by nucleotide sequencing with corresponding primers. Two-hybrid screening was carried out based on the protocol described in Two-Hybrid System Manual (Invitrogen, USA). The result of screening was further confirmed by the two-hybrid assay. The full-length CDS of *PQT3* and the four segments of *PQT3* were inserted into pDEST32 vector to construct the bait plasmid, while the prey plasmid was constructed with pDEST22 vector and the full-length CDS of *PRMT4b*. For two-hybrid assay, the primers could be found in S1 Table.

### Pull-down assay

MBP-PQT3-C66 and His-AtPRMT4b fusion protein were expressed using prokaryotic expression system and purified. MBP-PQT3-C66 fusion protein was incubated with MBP beads (amylose resin) at 4°C for 2 h, and the MBP tag was used as a negative control. The beads were cleaned with washing buffer for 4 times. Then the beads were incubated with His-AtPRMT4b at 4°C for 2 h respectively. The beads were cleaned with washing buffer for 4 times. Western blot was used to detect the SDS-PAGE separation results of pulled-down mixtures in nitrocellulose membrane with anti-His antibody.

### Agroinfiltration procedure

*Agrobacterium tumefaciens* strain C58C1 was used in the experiments. Agroinfiltration procedure was carried out as described previously [63]. At first, these strains were grown on LB medium with Gentamicin and Kanamycin. Single colony was transferred into 5 ml LB liquid medium containing same resistance and grown for 48 h in a 28°C shaker. The bacteria solution was inoculated into new LB liquid medium containing 40 μM acetosyringone (1:100 ratio, v/v) and 10 mM 2-(N-morpholine)-ethanesulfonic acid (MES; pH 5.6). Bacteria were developed in a 28°C shaker until OD_600_ reached 3.0 approximately. The bacteria were collected gently by means of 10 min centrifugation (3,200 g/min), and the resuspension of pellets was performed with 10 mM MgCl_2_ until OD_600_ reached 1.5 approximately. The bacteria solution was kept at room temperature with a final concentration of 200 μM acetosyringone for at least 3 h without shaking. The different plastid combinations were transformed into epidermal cells of *N*. *benthamiana* leaves by disposable syringe.

### BiFC analysis

NE-PQT3 (the N-terminus of YFP fused with PQT3) and CE-PRMT4b (the C-terminus of YFP fused with PRMT4b) were constructed. These constructs were transferred to Agrobacterium strains C58C1 respectively. As mentioned above, the different plastid combinations were transformed into epidermal cells of *N*. *benthamiana* leaves by agroinfiltration. YFP was observed 1–2 days after leaf infiltration using confocal. The nuclei were stained by Hoechst subsequently and fluorescence detection by confocal was performed. For BiFC assay, the primers could be found in S1 Table.

### Protein extraction

Native extraction buffer 1 [10 mM EDTA; 50 mM TRIS-MES, pH 8.0; 1 mM MgCl_2_; 5 mM DTT; 0.5 M sucrose; protease inhibitor cocktail for plant cell and tissue extracts (Sigma, USA)] was chosen for protein extraction buffer. Other steps of protein extraction were carried out as described previously [63].

### *In planta* ubiquitination assay

*N*. *benthamiana* leaves was infiltrated using the mixture of Agrobacterium strains containing different constructs, from which total proteins were extracted 1 day after Agroinfiltration. The p19 was used as gene-silencing suppressor. The anti-HA antibody was used for western blot detection of the total proteins after the separation of SDS-PAGE. HA-PRMT4b is a fusion protein composed of HA-tag and PRMT4b.The protein level of PRMT4b could be analyzed via the HA-tag detection using anti-HA antibody. Ponceau S staining of the Rubisco protein was used as loading control. In the presence of MG132, total proteins were immunoprecipitated with protein A agarose beads (Millipore, USA) and anti-HA antibody (Abmart) subsequently. Detailed steps of immunoprecipitation were carried out as described previously [63]. Immunoprecipitated samples were analyzed using western blot with anti-ubiquitin antibody (Abcam). Image Quant LAS 4000 (GE, USA), as the CCD camera system, was used for the result examination.

### Chromatin immunoprecipitation-PCR assay

The wild type, *pqt3* mutant, *prmt4b* mutant and *pqt3prmt4b* double mutants were used for ChIP assay without or with 6 μM paraquat treatment for 24h. *UBQ5* was chose for internal control. ChIP was performed as previously described [97, 98]. The regions with Arg-17 methylation were precipitated via anti-H3R17me2a antibodies (anti-Histone H3 asymmetric dimethyl R17 antibody-ChIP grade, ab8284, Abcam, USA) from input DNA. The corresponding primers were designed for quantitative RT-PCR to detect the enrichments of different DNA fragments in *APX1* and *GPX1* chromatins [99, 100]. The primers used in ChIP assay were showed in S1 Table.

## Supporting Information

S1 Fig Identification of mutants, homozygous Salk lines and transgenic lines. **(A)** The location of the T-DNA insertions in *pqt3-1* mutant and *pqt3-2* mutant. (Salk_065409). The locations of T-DNA insertion were shown as inverted black triangles. The structure of the *At4g17410* locus was shown for exons as red boxes, introns as black lines and UTR as black box. **(B)** Detection of transcript levels for *PQT3* and its neighboring genes using RT-PCR. The transcript levels of *At4g17390*, *At4g17410* (*PQT3*), and *At4g17420* were compared between wild type and *pqt3-1* mutant. *Tubulin8* (*TUB8*) was used as a loading control. The RT-PCR assay was repeated for three times, and a typical result was shown. **(C)** Genomic PCR analysis of homozygous Salk_065409. Genomic DNA isolated from leaves of Salk_065409 line and wild type was used as template for PCR. **(D)** RT-PCR analysis of *pqt3-1* mutant and homozygous T-DNA insertion mutant of Salk_ 065409 (*pqt3-2*). RNA was extracted from 2-week-old wild type, *pqt3-1* mutant and Salk_065409 (*pqt3-2*). The transcript level of *PQT3* was analyzed by RT-PCR. No signal was detected in *pqt3-1* mutant and the homozygous Salk_065409 (*pqt3-2*). *TUB8* was used as a loading control. **(E)** RT-PCR analysis of *PQT3* transcript levels using RNA samples isolated from wild type, *35Spro*:*PQT3*, and FC line. **(F)** Identification of *35Spro*:*PQT3* using quantitative RT-PCR. RNA was extracted from 4-week-old wild type and *35Spro*:*PQT3* lines. Values are mean ± SD (n = 3 experiments, *P < 0.05). Asterisk indicate Student’s t-test significant difference. **(G)** Genomic PCR analysis of homozygous Salk_097442C (*prmt4b*). Genomic DNA isolated from leaves of Salk_097442C line and wild type was used as template for PCR. **(H)** quantitative RT-PCR analysis of *prmt4b* mutant (Salk_097442C). RNA was extracted from 4-week-old wild type and *prmt4b* mutant. No signal was detected in *prmt4b* mutant. **(I)** Genomic PCR analysis of homozygous Salk_033423 (*prmt4a*). Genomic DNA isolated from leaves of Salk_033423 line and wild type was used as template for PCR. **(J and K)** Genomic PCR analysis of *prmt4aprmt4b* double mutants. Genomic DNA isolated from leaves of *prmt4aprmt4b* double mutants and wild type was used as template for PCR.The knockout of *PRMT4a* **(J)** and *PRMT4b* **(K)** was identified respectively. **(L)** Identification of *35Spro*:*PRMT4b* using quantitative RT-PCR. RNA was extracted from 4-week-old wild type and *35Spro:PRMT4b* lines. Values are mean ± SD (n = 3 experiments, **P < 0.01, ***P < 0.001). Asterisks indicate Student’s t-test significant differences. **(M and N)** Identification of *pqt3prmt4a* double mutants. The knockout of *PRMT4a* in *pqt3prmt4a* double mutants was analyzed by Genomic PCR **(M)**. The transcript level of *PQT3* was detected by RT-PCR **(N)**. No signal was detected in *pqt3prmt4a* double mutants. *TUB8* was used as a loading control. **(O and P)** Identification of *pqt3prmt4b* double mutants. The knockout of *PRMT4b* in *pqt3prmt4b* double mutants was analyzed by Genomic PCR **(O)**. The transcript level of *PQT3* was detected by RT-PCR **(P)**. No signal was detected in *pqt3prmt4b* double mutants. *TUB8* was used as a loading control.
(DOCX)

S2 Fig Phenotype of *pqt3* mutants under other environmental stresses lead to oxidative damage. **(A)** The phenotype of wild type, *pqt3-1* and *pqt3-2* mutant grown on MS medium for 12 days. Bar = 1 cm. **(B)** Primary root elongation of wild type, *pqt3-1* and *pqt3-2* mutant grown on MS medium for 12 days was measured. Values are mean ± SD (n = 30 plants). **(C)** The phenotype of wild type, *pqt3-1* and *pqt3-2* mutant grown on MS medium with 150 μM CdCl_2_ for 12 days. Bar = 1 cm. **(D)** Primary root elongation of wild type, *pqt3-1* and *pqt3-2* mutant grown on MS with 150 μM CdCl_2_ for 12 days was measured. Values are mean ± SD (n = 30 plants, **P < 0.01). Asterisks indicate Student’s t-test significant differences. **(E)** The phenotype of wild type, *pqt3-1* and *pqt3-2* mutant grown on MS medium containing 250 mM mannitol for 12 days. Bar = 1 cm. **(F)** Primary root elongation of wild type, *pqt3-1* and *pqt3-2* mutant grown on MS containing 250 mM mannitol for 12 days was measured. Values are mean ± SD (n = 30 plants, **P < 0.01, ***P < 0.001). Asterisks indicate Student’s t-test significant differences. **(G)** The phenotype of wild type, *pqt3-1* and *pqt3-2* mutant grown on MS medium with 120 mM NaCl for 12 days. Bar = 1 cm. **(H)** Primary root elongation of wild type, *pqt3-1* and *pqt3-2* mutant grown on MS with 120 mM NaCl for 12 days was measured. Values are mean ± SD (n = 30 plants, *P < 0.05, **P < 0.01). Asterisks indicate Student’s t-test significant differences. **(I)** The schematic diagram of the locations of wild type, *pqt3-1* and *pqt3-2* plants grown in one pot for drought tolerance assay. **(J to L)** Drought stress assay of the *pqt3* mutants and wild type grown in the same pot. The wild type, *pqt3-1*, and *pqt3-2* plants were grown in the same pot for 15 days before drought stress was imposed. These plants were grown under drought stress for 15 days. The photos were taken before re-watering **(J)** and after re-watering for 1 day **(K)** and 7 days **(L)**. **(M)** Re-water survival ratio of wild type, *pqt3-1* and *pqt3-2* mutant after drought stress was counted. Values are mean ± SD (n = 18 plants, ***P < 0.001). Asterisks indicate Student’s t-test significant differences.
(DOCX)

S3 Fig Sections of PQT3 protein used in Y2H, pull-down and *in vitro* ubiquitination assay. **(A)** The division of PQT3 protein for Y2H assay was presented by the red lines. The full-length protein sequence of PQT3 was divided into four segments (DWNN, zfCCHC, U-box/RING finger and C-terminus contained the NLS1 and NLS2 domain) and each segment was used as bait. In pull-down assay, the protein section of PQT3 (PQT3-C66) located in the blue box was used. **(B)** The protein used in Self-ubiquitin assay. Full-length protein sequence of PQT3 and PQT3-N40 (the section of PQT3 protein located in the red box) were selected for the assay. PQT3-N40 (1–360 aa) contains all the conserved domains of an E3 ubiquitin ligase.
(DOCX)

S4 Fig The transcript level of *PQT3* after the elimination of PQ stress. 1-week-old wild type seedlings were treated by 6 μM paraquat for 3h. PQ stress diminished for 3h subsequently before RNA was extracted for quantitative RT-PCR analysis. Values are mean ± SD (n = 3 experiments, ***P < 0.001). Asterisks indicate Student’s t-test significant differences.
(DOCX)

S5 Fig Y2H assay for PQT3 and PRMT4a. PQT3 and its four protein sections were used as the bait. PRMT4a was used as the prey. Krev1/RalGDS-wt act as strong positive control and Krev1/RalGDS-m1 act as week positive control. Krev1/RalGDS-m2 was used for negative control. The yeast harboring various constructs was grown on SD-Leu-Trp medium (upper panel). The yeast was transferred to SD-Leu-Trp-His medium with 50 mM 3-AT (middle panel) or used for X-gal staining (lower panel).
(DOCX)

S6 Fig The mRNA level of *PRMT4b* in wild type and *pqt3* mutant under paraquat treatment. Total RNA was isolated from 14-day-old wild type and *pqt3* seedlings without or with 6 μM paraquat treatment for quantitative RT-PCR analysis. Values are mean ± SD (n = 3 experiments).
(DOCX)

S7 Fig Phenotype of *PQT3* and *PRMT4b* overexpression lines under other environmental stresses lead to oxidative damage. **(A)** The phenotype of wild type, *35S*:*PRMT4b-1*, *35S*:*PRMT4b-2*, *35S*:*PQT3-1*, and *35S*:*PQT3-2* grown on MS medium for 14 days. Bar = 1 cm. **(B)** Primary root elongation of wild type, *35S*:*PRMT4b-1*, *35S*:*PRMT4b-2*, *35S*:*PQT3-1*, and *35S*:*PQT3-2* grown on MS for 14 days was measured. Values are mean ± SD (n = 30 plants). **(C)** The phenotype of wild type, *35S*:*PRMT4b-1*, *35S*:*PRMT4b-2*, *35S*:*PQT3-1*, and *35S*:*PQT3-2* grown on MS medium with 150 μM CdCl_2_ for 14 days. Bar = 1 cm. **(D)** Primary root elongation of wild type, *35S*:*PRMT4b-1*, *35S*:*PRMT4b-2*, *35S*:*PQT3-1*, and *35S*:*PQT3-2* grown on MS with 150 μM CdCl_2_ for 14 days was measured. Values are mean ±SD (n = 30 plants, *P < 0.05). Asterisk indicate Student’s t-test significant difference. **(E)** The phenotype of wild type, *35S*:*PRMT4b-1*, *35S*:*PRMT4b-2*, *35S*:*PQT3-1*, and *35S*:*PQT3-2* grown on MS medium with 150 mM NaCl for 14 days. Bar = 1 cm. **(F)** Primary root elongation of wild type, *35S*:*PRMT4b-1*, *35S*:*PRMT4b-2*, *35S*:*PQT3-1*, and *35S*:*PQT3-2* grown on MS with 150 mM NaCl for 14 days was measured. Values are mean ± SD (n = 30 plants, *P < 0.05). Asterisk indicate Student’s t-test significant difference.
(DOCX)

S8 Fig The analysis of antioxidant enzyme genes in wild type, *prmt4b* mutant, and *35Spro*:*PRMT4b*. **(A-J)** Quantitative RT-PCR analysis of transcript levels of antioxidant enzyme genes. RNA samples were isolated from 7-day-old wild type, *prmt4b* mutant and *35S*:*PRMT4b* seedlings for quantitative RT-PCR analysis. The transcript levels of APX **(A)**, GPX **(B)**, ACHT **(C)**, FSD **(D)**, CAT **(E)**, GRXC **(F)**, CSD **(G)**, PRXQ **(H)**, 2CPB **(I)** and MSD **(J)** were analyzed. Values are mean ± SD (n = 3 experiments, *P < 0.05, **P < 0.01, ***P < 0.001). Asterisks indicate Student’s t-test significant differences.
(DOCX)

S9 Fig Functional domains and phylogenetic tree of PQT3 protein. **(A)** The conserved domains and NLSs (nuclear localization signals) of PQT3 protein. Based on predicted secondary structure with InterProScan protein sequence analysis software, the functional domains were marked using Vector NTI Advance software of Invitrogen. **(B)** Functional domains and the corresponding protein sequence of PQT3. Purple mark represents DWNN domain (amino acids 3–78); dark yellow mark represents Zinc finger (C2HC) domain (amino acids 201–218), and the red amino acids represent the conserved cysteine (C) and histidine (H); pink mark represents RING-finger (C6HC2) domain (amino acids 288–326), and the white amino acids represent the conserved cysteine (C) and histidine (H); yellow mark, including pink mark, represents U-box domain (amino acids 280–356); Two blue marks in C-terminus represent two NLS domains (amino acids 471–477 and 696–711). **(C)** Phylogenetic tree of PQT3 protein. AT4G17410: *Arabidopsis thaliana* PQT3; Bo_ABD64942.1, Bo_ABD65123.1: *Brassica oleracea*; Pt_XP_002326651.1: *Populus trichocarpa*; Rc_XP_002530663.1: *Ricinus communis*; Vv_CBI23464.3: *Vitis vinifera*; OsI_EEC67002.1: *Oryza sativa Indica Group*; OsJ_EEE50995.1: *Oryza sativa Japonica Group*. The result showed that the highest homology protein of Arabidopsis PQT3 is Bo_ABD65123.1 in *Brassica oleracea*.
(DOCX)

S10 Fig Homologous sequences alignment of PQT3 protein. DWNN domain (3–78) and RING-finger/U-box domain (295–371) were conserved in different plants. Yellow shading indicates the same sequence of different plant proteins as PQT3; blue shading indicates the conserved sequence; green shading indicates the block similar sequence and white shading indicates the weak similar sequence (green word) and different sequence (black word).
(DOCX)

S1 Table The primers used in this study. (DOCX)
