# Supplementary material for: PARAQUAT TOLERANCE3 Is an E3 Ligase That Switches off Activated Oxidative Response by Targeting Histone-Modifying PROTEIN METHYLTRANSFERASE4b
Source: PLoS Genet. 2016 Sep 27;12(9):e1006332. doi: 10.1371/journal.pgen.1006332 (PMC5038976; doi:10.1371/journal.pgen.1006332)
Supplement: S1 Fig — (A) The location of the T-DNA insertions in pqt3-1 mutant and pqt3-2 mutant. (Salk_065409). The locations of T-DNA insertion were shown as inverted black triangles. The structure of the At4g17410 locus was shown for exons as red boxes, introns as black lines and UTR as black box. (B) Detection of transcript levels for PQT3 and its neighboring genes using RT-PCR. The transcript levels of At4g17390, At4g17410 (PQT3), and At4g17420 were compared between wild type and pqt3-1 mutant. Tubulin8 (TUB8) was used as a loading control. The RT-PCR assay was repeated for three times, and a typical result was shown. (C) Genomic PCR analysis of homozygous Salk_065409. Genomic DNA isolated from leaves of Salk_065409 line and wild type was used as template for PCR. (D) RT-PCR analysis of pqt3-1 mutant and homozygous T-DNA insertion mutant of Salk_ 065409 (pqt3-2). RNA was extracted from 2-week-old wild type, pqt3-1 mutant and Salk_065409 (pqt3-2). The transcript level of PQT3 was analyzed by RT-PCR. No signal was detected in pqt3-1 mutant and the homozygous Salk_065409 (pqt3-2). TUB8 was used as a loading control. (E) RT-PCR analysis of PQT3 transcript levels using RNA samples isolated from wild type, 35Spro:PQT3, and FC line. (F) Identification of 35Spro:PQT3 using quantitative RT-PCR. RNA was extracted from 4-week-old wild type and 35Spro:PQT3 lines. Values are mean ± SD (n = 3 experiments, *P < 0.05). Asterisk indicate Student’s t-test significant difference. (G) Genomic PCR analysis of homozygous Salk_097442C (prmt4b). Genomic DNA isolated from leaves of Salk_097442C line and wild type was used as template for PCR. (H) quantitative RT-PCR analysis of prmt4b mutant (Salk_097442C). RNA was extracted from 4-week-old wild type and prmt4b mutant. No signal was detected in prmt4b mutant. (I) Genomic PCR analysis of homozygous Salk_033423 (prmt4a). Genomic DNA isolated from leaves of Salk_033423 line and wild type was used as template for PCR. (J and K) Genomic PCR analysis of prmt4apr [file pgen.1006332.s001.docx]

**Supporting Information for "PARAQUAT TOLERANCE3 is an E3 ligase that switches off activated oxidative response by targeting histone-modifying PROTEIN METHYLTRANSFERASE4b" by Luo et al.**


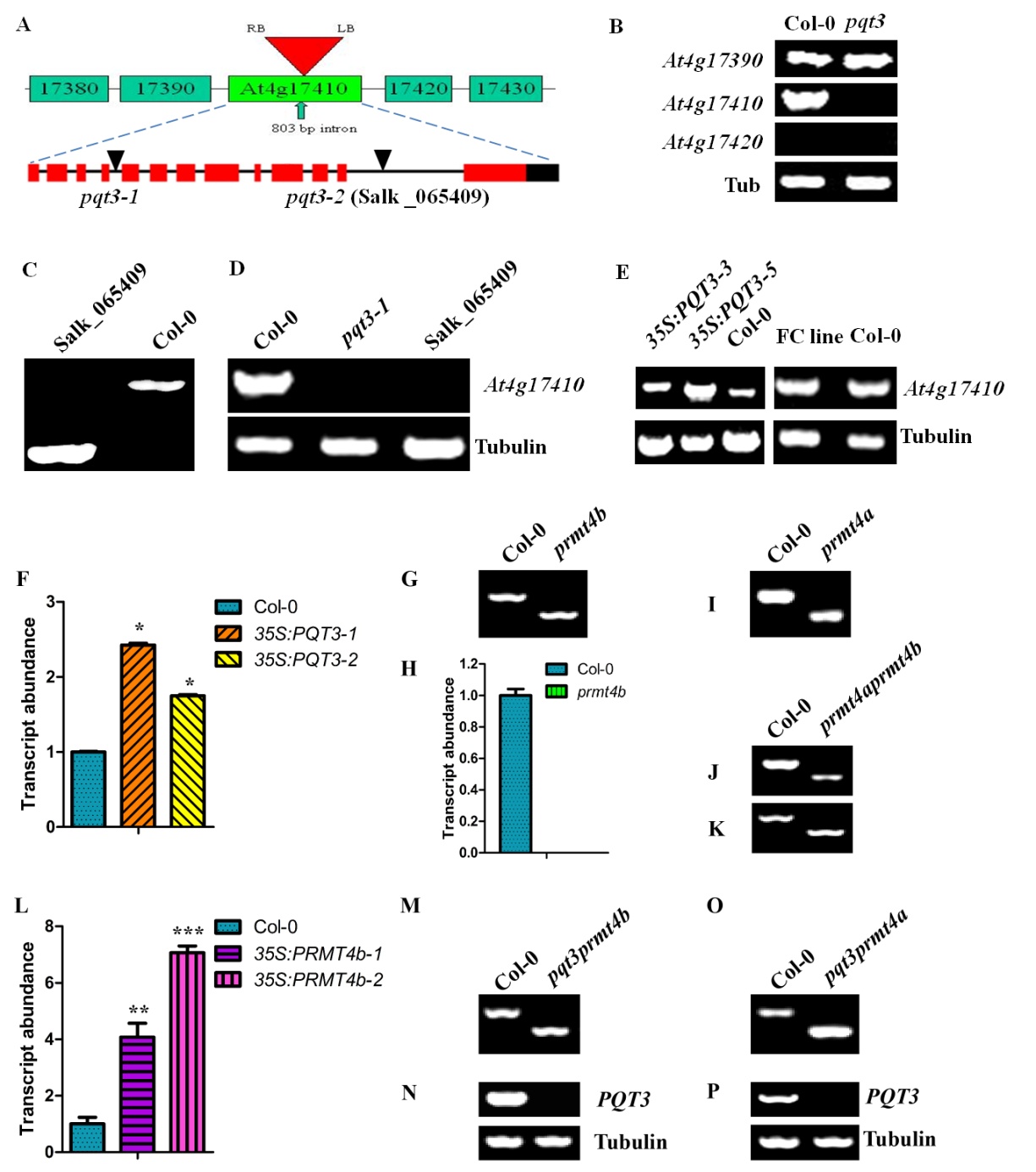


**S1 Fig. Identification of mutants, homozygous Salk lines and transgenic lines.**

**(A)** The location of the T-DNA insertions in *pqt3-1* mutant and *pqt3-2* mutant (Salk_065409). The locations of T-DNA insertion were shown as inverted black triangles. The structure of the *At4g17410* locus was shown for exons as red boxes, introns as black lines and UTR as black box.

**(B)** Detection of transcript levels for *PQT3* and its neighboring genes using RT-PCR. The transcript levels of *At4g17390*, *At4g17410* (*PQT3*), and *At4g17420* were compared between wild type and *pqt3-1* mutant. *Tubulin8* (*TUB8*) was used as a loading control. The RT-PCR assay was repeated for three times, and a typical result was shown.

**(C)** Genomic PCR analysis of homozygous Salk_065409. Genomic DNA isolated from leaves of Salk_065409 line and wild type was used as template for PCR.

**(D)** RT-PCR analysis of *pqt3-1* mutant and homozygous T-DNA insertion mutant of Salk_ 065409 (*pqt3-2*). RNA was extracted from 2-week-old wild type, *pqt3-1* mutant and Salk_065409 (*pqt3-2*). The transcript level of *PQT3* was analyzed by RT-PCR. No signal was detected in *pqt3-1* mutant and the homozygous Salk_065409 (*pqt3-2*). *TUB8* was used as a loading control.

**(E)** RT-PCR analysis of *PQT3* transcript levels using RNA samples isolated from wild type, *35Spro:PQT3,* and FC line.

**(F)** Identification of *35Spro:PQT3* using quantitative RT-PCR. RNA was extracted from 4-week-old wild type and *35Spro:PQT3* lines. Values are mean ±SD (n=3 experiments, *P < 0.05). Asterisk indicate Student’s t-test signiﬁcant difference.

**(G)** Genomic PCR analysis of homozygous Salk_097442C (*prmt4b*). Genomic DNA isolated from leaves of Salk_097442C line and wild type was used as template for PCR.

**(H)** quantitative RT-PCR analysis of *prmt4b* mutant (Salk_097442C). RNA was extracted from 4-week-old wild type and *prmt4b* mutant. No signal was detected in *prmt4b* mutant.

**(I)** Genomic PCR analysis of homozygous Salk_033423 (*prmt4a*). Genomic DNA isolated from leaves of Salk_033423 line and wild type was used as template for PCR.

**(J and K)** Genomic PCR analysis of *prmt4aprmt4b* double mutants. Genomic DNA isolated from leaves of *prmt4aprmt4b* double mutants and wild type was used as template for PCR.The knockout of *PRMT4a* **(J)** and *PRMT4b* **(K)** was identified respectively.

**(L)** Identification of *35Spro:PRMT4b* using quantitative RT-PCR. RNA was extracted from 4-week-old wild type and *35Spro:PRMT4b* lines. Values are mean ±SD (n=3 experiments, **P < 0.01, ***P < 0.001). Asterisks indicate Student’s t-test signiﬁcant differences.

**(M and N)** Identification of *pqt3prmt4a* double mutants. The knockout of *PRMT4a* in *pqt3prmt4a* double mutants was analyzed by Genomic PCR **(M)**. The transcript level of *PQT3* was detected by RT-PCR **(N)**. No signal was detected in *pqt3prmt4a* double mutants. *TUB8* was used as a loading control.

**(O and P)** Identification of *pqt3prmt4b* double mutants. The knockout of *PRMT4b* in *pqt3prmt4b* double mutants was analyzed by Genomic PCR **(O)**. The transcript level of *PQT3* was detected by RT-PCR **(P)**. No signal was detected in *pqt3prmt4b* double mutants. *TUB8* was used as a loading control.
