# Supplementary material for: PARAQUAT TOLERANCE3 Is an E3 Ligase That Switches off Activated Oxidative Response by Targeting Histone-Modifying PROTEIN METHYLTRANSFERASE4b
Source: PLoS Genet. 2016 Sep 27;12(9):e1006332. doi: 10.1371/journal.pgen.1006332 (PMC5038976; doi:10.1371/journal.pgen.1006332)
Supplement: S2 Fig — (A) The phenotype of wild type, pqt3-1 and pqt3-2 mutant grown on MS medium for 12 days. Bar = 1 cm. (B) Primary root elongation of wild type, pqt3-1 and pqt3-2 mutant grown on MS medium for 12 days was measured. Values are mean ± SD (n = 30 plants). (C) The phenotype of wild type, pqt3-1 and pqt3-2 mutant grown on MS medium with 150 μM CdCl2 for 12 days. Bar = 1 cm. (D) Primary root elongation of wild type, pqt3-1 and pqt3-2 mutant grown on MS with 150 μM CdCl2 for 12 days was measured. Values are mean ± SD (n = 30 plants, **P < 0.01). Asterisks indicate Student’s t-test significant differences. (E) The phenotype of wild type, pqt3-1 and pqt3-2 mutant grown on MS medium containing 250 mM mannitol for 12 days. Bar = 1 cm. (F) Primary root elongation of wild type, pqt3-1 and pqt3-2 mutant grown on MS containing 250 mM mannitol for 12 days was measured. Values are mean ± SD (n = 30 plants, **P < 0.01, ***P < 0.001). Asterisks indicate Student’s t-test significant differences. (G) The phenotype of wild type, pqt3-1 and pqt3-2 mutant grown on MS medium with 120 mM NaCl for 12 days. Bar = 1 cm. (H) Primary root elongation of wild type, pqt3-1 and pqt3-2 mutant grown on MS with 120 mM NaCl for 12 days was measured. Values are mean ± SD (n = 30 plants, *P < 0.05, **P < 0.01). Asterisks indicate Student’s t-test significant differences. (I) The schematic diagram of the locations of wild type, pqt3-1 and pqt3-2 plants grown in one pot for drought tolerance assay. (J to L) Drought stress assay of the pqt3 mutants and wild type grown in the same pot. The wild type, pqt3-1, and pqt3-2 plants were grown in the same pot for 15 days before drought stress was imposed. These plants were grown under drought stress for 15 days. The photos were taken before re-watering (J) and after re-watering for 1 day (K) and 7 days (L). (M) Re-water survival ratio of wild type, pqt3-1 and pqt3-2 mutant after drought stress was counted. Values are mean ± SD (n = 18 plants, ***P < 0.001). Asterisks i [file pgen.1006332.s002.docx]

**Supporting Information for "PARAQUAT TOLERANCE3 is an E3 ligase that switches off activated oxidative response by targeting histone-modifying PROTEIN METHYLTRANSFERASE4b" by Luo et al.**


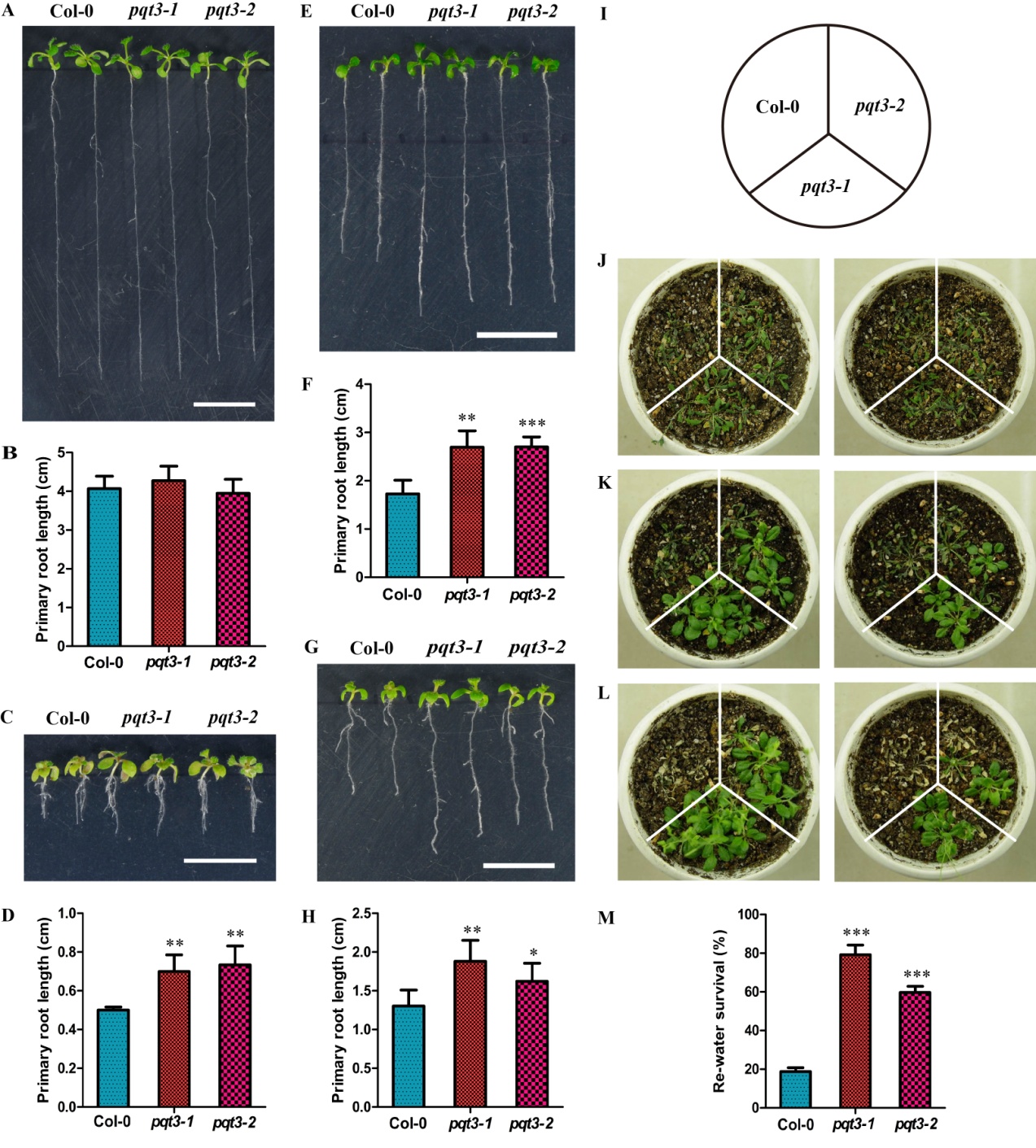


**S2 Fig. Phenotype of *pqt3* mutants under other environmental stresses lead to oxidative damage.**

**(A)** The phenotype of wild type, *pqt3-1* and *pqt3-2* mutant grown on MS medium for 12 days. Bar= 1 cm.

**(B)** Primary root elongation of wild type, *pqt3-1* and *pqt3-2* mutant grown on MS medium for 12 days was measured. Values are mean ±SD (n= 30 plants).

**(C)** The phenotype of wild type, *pqt3-1* and *pqt3-2* mutant grown on MS medium with 150 μM CdCl_2_ for 12 days. Bar= 1 cm.

**(D)** Primary root elongation of wild type, *pqt3-1* and *pqt3-2* mutant grown on MS with 150 μM CdCl_2_ for 12 days was measured. Values are mean ±SD (n= 30 plants, **P < 0.01). Asterisks indicate Student’s t-test signiﬁcant differences.

**(E)** The phenotype of wild type, *pqt3-1* and *pqt3-2* mutant grown on MS medium containing 250 mM mannitol for 12 days. Bar= 1 cm.

**(F)** Primary root elongation of wild type, *pqt3-1* and *pqt3-2* mutant grown on MS containing 250 mM mannitol for 12 days was measured. Values are mean ±SD (n= 30 plants, **P < 0.01, ***P < 0.001). Asterisks indicate Student’s t-test signiﬁcant differences.

**(G)** The phenotype of wild type, *pqt3-1* and *pqt3-2* mutant grown on MS medium with 120 mM NaCl for 12 days. Bar= 1 cm.

**(H)** Primary root elongation of wild type, *pqt3-1* and *pqt3-2* mutant grown on MS with 120 mM NaCl for 12 days was measured. Values are mean ±SD (n= 30 plants, *P < 0.05, **P < 0.01). Asterisks indicate Student’s t-test signiﬁcant differences.

**(I)** The schematic diagram of the locations of wild type, *pqt3-1* and *pqt3-2* plants grown in one pot for drought tolerance assay.

**(J to L)** Drought stress assay of the *pqt3* mutants and wild type grown in the same pot. The wild type, *pqt3-1*, and *pqt3-2* plants were grown in the same pot for 15 days before drought stress was imposed. These plants were grown under drought stress for 15 days. The photos were taken before re-watering **(J)** and after re-watering for 1 day **(K)** and 7 days **(L)**.

**(M)** Re-water survival ratio of wild type, *pqt3-1* and *pqt3-2* mutant after drought stress was counted. Values are mean ±SD (n= 18 plants, ***P < 0.001). Asterisks indicate Student’s t-test signiﬁcant differences.
