# Supplementary material for: PARAQUAT TOLERANCE3 Is an E3 Ligase That Switches off Activated Oxidative Response by Targeting Histone-Modifying PROTEIN METHYLTRANSFERASE4b
Source: PLoS Genet. 2016 Sep 27;12(9):e1006332. doi: 10.1371/journal.pgen.1006332 (PMC5038976; doi:10.1371/journal.pgen.1006332)
Supplement: S3 Fig — (A) The division of PQT3 protein for Y2H assay was presented by the red lines. The full-length protein sequence of PQT3 was divided into four segments (DWNN, zfCCHC, U-box/RING finger and C-terminus contained the NLS1 and NLS2 domain) and each segment was used as bait. In pull-down assay, the protein section of PQT3 (PQT3-C66) located in the blue box was used. (B) The protein used in Self-ubiquitin assay. Full-length protein sequence of PQT3 and PQT3-N40 (the section of PQT3 protein located in the red box) were selected for the assay. PQT3-N40 (1–360 aa) contains all the conserved domains of an E3 ubiquitin ligase. (DOCX) [file pgen.1006332.s003.docx]

**Supporting Information for "PARAQUAT TOLERANCE3 is an E3 ligase that switches off activated oxidative response by targeting histone-modifying PROTEIN METHYLTRANSFERASE4b" by Luo et al.**


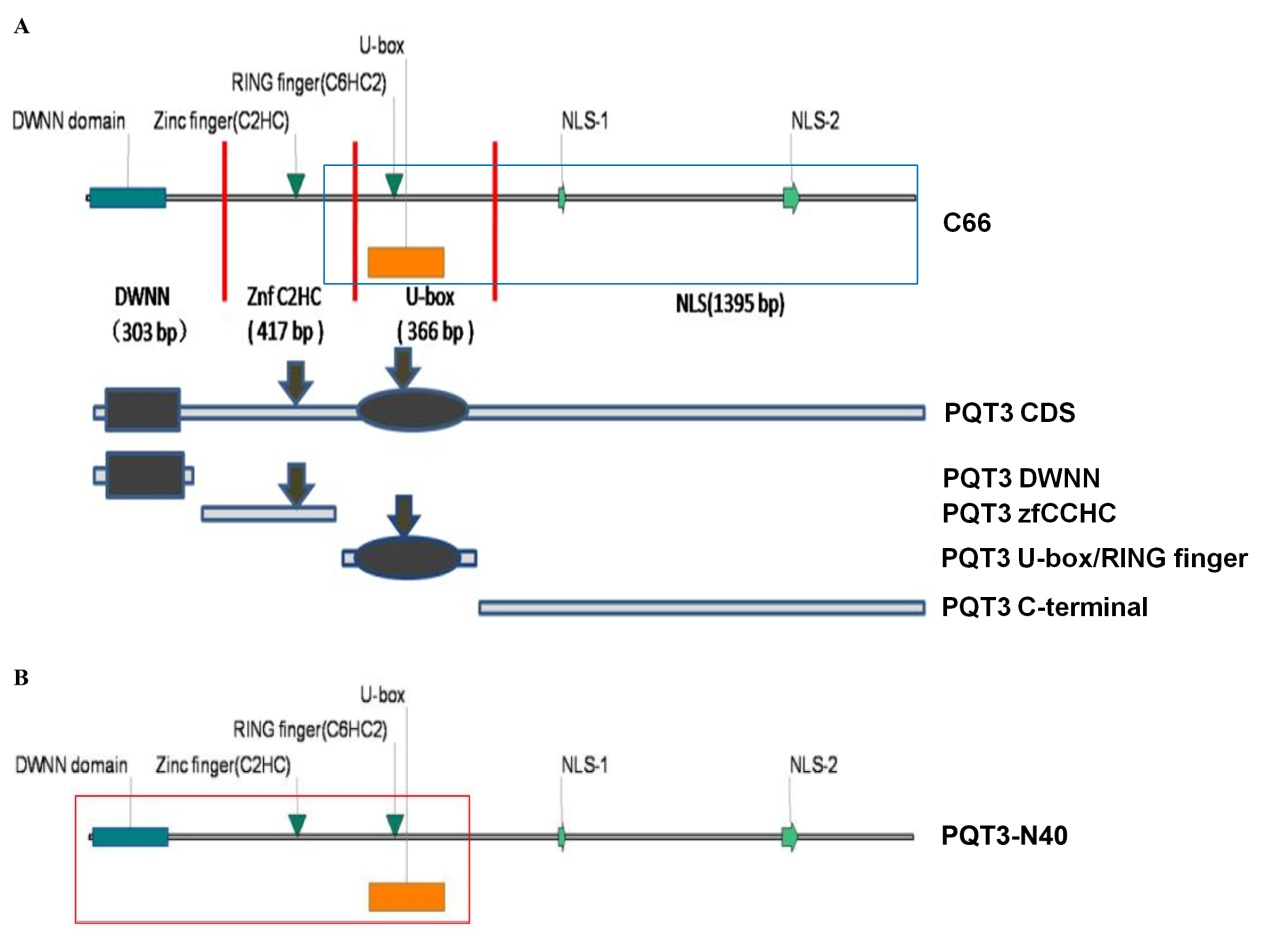


**S3 Fig. Sections of PQT3 protein used in Y2H, pull-down and *in vitro* ubiquitination assay.**

**(A)** The division of PQT3 protein for Y2H assay was presented by the red lines. The full-length protein sequence of PQT3 was divided into four segments (DWNN, zfCCHC, U-box/RING finger and C-terminus contained the NLS1 and NLS2 domain) and each segment was used as bait. In pull-down assay, the protein section of PQT3 (PQT3-C66) located in the blue box was used.

**(B)** The protein used in Self-ubiquitin assay. Full-length protein sequence of PQT3 and PQT3-N40 (the section of PQT3 protein located in the red box) were selected for the assay. PQT3-N40 (1-360 aa) contains all the conserved domains of an E3 ubiquitin ligase.
