# Supplementary material for: PARAQUAT TOLERANCE3 Is an E3 Ligase That Switches off Activated Oxidative Response by Targeting Histone-Modifying PROTEIN METHYLTRANSFERASE4b
Source: PLoS Genet. 2016 Sep 27;12(9):e1006332. doi: 10.1371/journal.pgen.1006332 (PMC5038976; doi:10.1371/journal.pgen.1006332)
Supplement: S4 Fig — 1-week-old wild type seedlings were treated by 6 μM paraquat for 3h. PQ stress diminished for 3h subsequently before RNA was extracted for quantitative RT-PCR analysis. Values are mean ± SD (n = 3 experiments, ***P < 0.001). Asterisks indicate Student’s t-test significant differences. (DOCX) [file pgen.1006332.s004.docx]

**Supporting Information for "PARAQUAT TOLERANCE3 is an E3 ligase that switches off activated oxidative response by targeting histone-modifying PROTEIN METHYLTRANSFERASE4b" by Luo et al.**


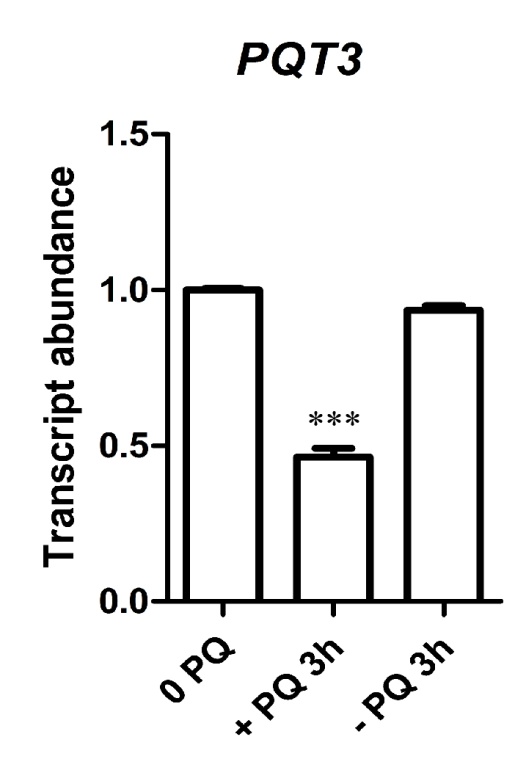


**S4 Fig. The transcript level of *PQT3* after the elimination of PQ stress.**

1-week-old wild type seedlings were treated with 6 μM paraquat for 3h. PQ stress diminished for 3h subsequently before RNA was extracted for quantitative RT-PCR analysis. Values are mean ± SD (n =3 experiments, ***P < 0.001). Asterisks indicate Student’s t-test signiﬁcant differences.
