# Supplementary material for: PARAQUAT TOLERANCE3 Is an E3 Ligase That Switches off Activated Oxidative Response by Targeting Histone-Modifying PROTEIN METHYLTRANSFERASE4b
Source: PLoS Genet. 2016 Sep 27;12(9):e1006332. doi: 10.1371/journal.pgen.1006332 (PMC5038976; doi:10.1371/journal.pgen.1006332)
Supplement: S5 Fig — PQT3 and its four protein sections were used as the bait. PRMT4a was used as the prey. Krev1/RalGDS-wt act as strong positive control and Krev1/RalGDS-m1 act as week positive control. Krev1/RalGDS-m2 was used for negative control. The yeast harboring various constructs was grown on SD-Leu-Trp medium (upper panel). The yeast was transferred to SD-Leu-Trp-His medium with 50 mM 3-AT (middle panel) or used for X-gal staining (lower panel). (DOCX) [file pgen.1006332.s005.docx]

**Supporting Information for "PARAQUAT TOLERANCE3 is an E3 ligase that switches off activated oxidative response by targeting histone-modifying PROTEIN METHYLTRANSFERASE4b" by Luo et al.**


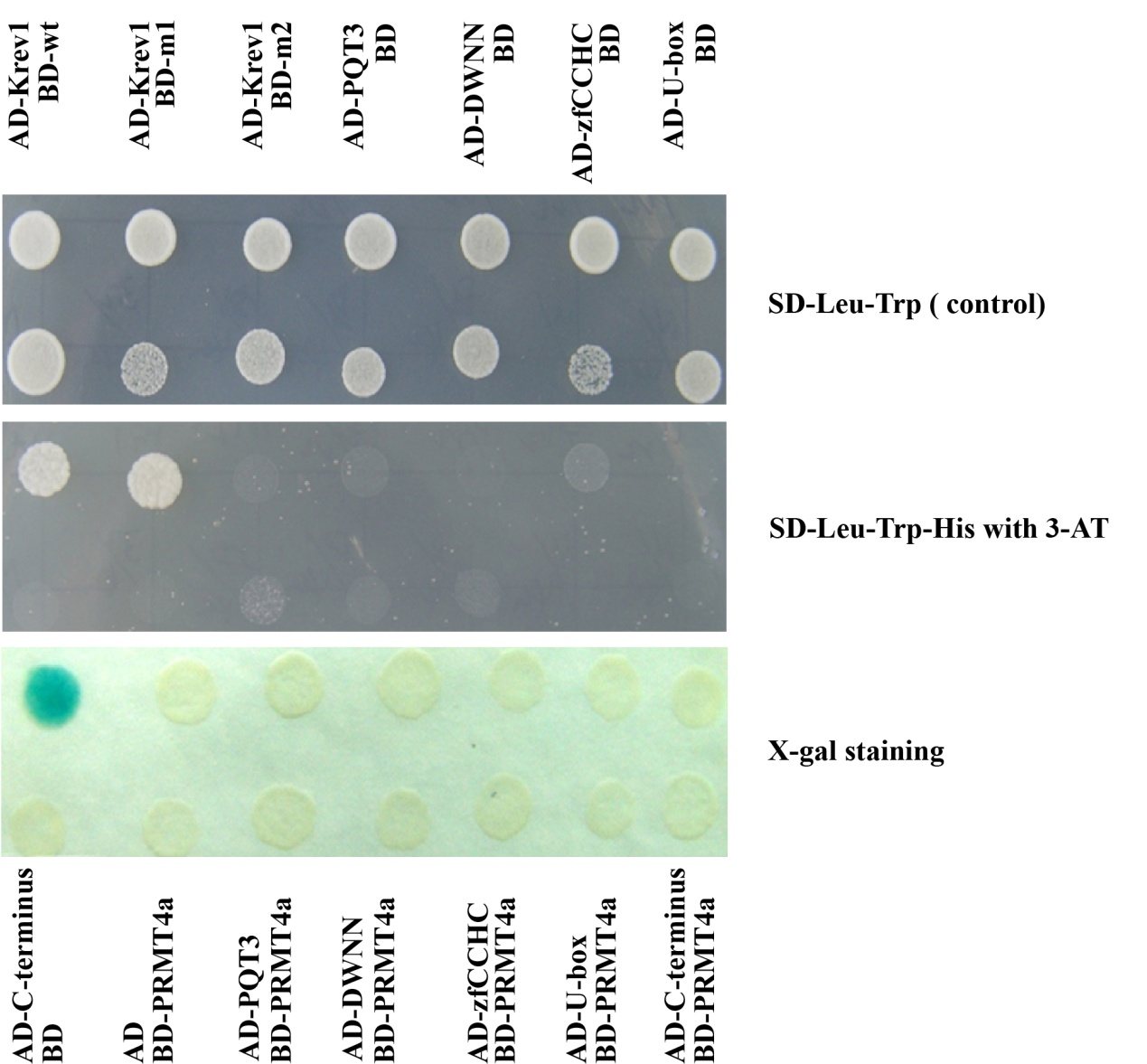


**S5 Fig. Y2H assay for PQT3 and PRMT4a.**

PQT3 and its four protein sections were used as the bait. PRMT4a was used as the prey. Krev1/RalGDS-wt act as strong positive control and Krev1/RalGDS-m1 act as week positive control. Krev1/RalGDS-m2 was used for negative control. The yeast harboring various constructs was grown on SC-Leu-Trp medium (upper panel). The yeast was transferred to SC-Leu-Trp-His medium with 50 mM 3-AT (middle panel) or used for X-gal staining (lower panel).
