# Supplementary material for: PARAQUAT TOLERANCE3 Is an E3 Ligase That Switches off Activated Oxidative Response by Targeting Histone-Modifying PROTEIN METHYLTRANSFERASE4b
Source: PLoS Genet. 2016 Sep 27;12(9):e1006332. doi: 10.1371/journal.pgen.1006332 (PMC5038976; doi:10.1371/journal.pgen.1006332)
Supplement: S6 Fig — Total RNA was isolated from 14-day-old wild type and pqt3 seedlings without or with 6 μM paraquat treatment for quantitative RT-PCR analysis. Values are mean ± SD (n = 3 experiments). (DOCX) [file pgen.1006332.s006.docx]

**Supporting Information for "PARAQUAT TOLERANCE3 is an E3 ligase that switches off activated oxidative response by targeting histone-modifying PROTEIN METHYLTRANSFERASE4b" by Luo et al.**

**
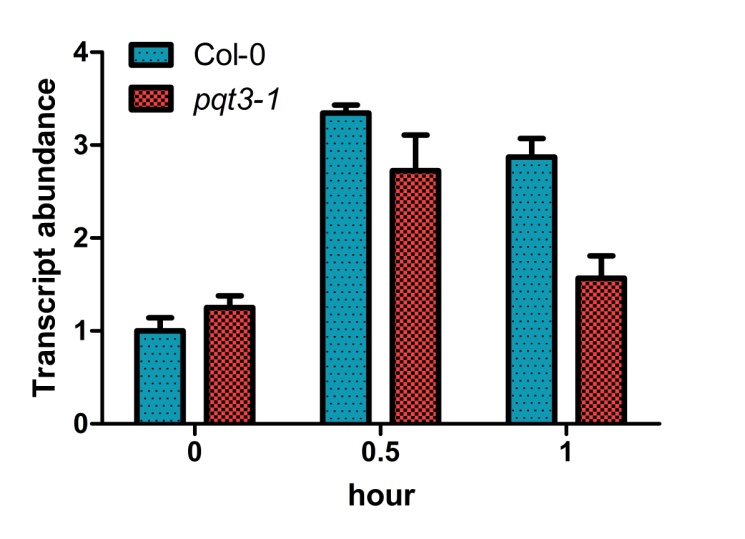
**

**S6 Fig. The mRNA level of *PRMT4b* in wild type and *pqt3* mutant under paraquat treatment.**

Total RNA was isolated from 14-day-old wild type and *pqt3* seedlings without or with 6 μM paraquat treatment for quantitative RT-PCR analysis. Values are mean ± SD (n=3 experiments).
