# Supplementary material for: PARAQUAT TOLERANCE3 Is an E3 Ligase That Switches off Activated Oxidative Response by Targeting Histone-Modifying PROTEIN METHYLTRANSFERASE4b
Source: PLoS Genet. 2016 Sep 27;12(9):e1006332. doi: 10.1371/journal.pgen.1006332 (PMC5038976; doi:10.1371/journal.pgen.1006332)
Supplement: S7 Fig — (A) The phenotype of wild type, 35S:PRMT4b-1, 35S:PRMT4b-2, 35S:PQT3-1, and 35S:PQT3-2 grown on MS medium for 14 days. Bar = 1 cm. (B) Primary root elongation of wild type, 35S:PRMT4b-1, 35S:PRMT4b-2, 35S:PQT3-1, and 35S:PQT3-2 grown on MS for 14 days was measured. Values are mean ± SD (n = 30 plants). (C) The phenotype of wild type, 35S:PRMT4b-1, 35S:PRMT4b-2, 35S:PQT3-1, and 35S:PQT3-2 grown on MS medium with 150 μM CdCl2 for 14 days. Bar = 1 cm. (D) Primary root elongation of wild type, 35S:PRMT4b-1, 35S:PRMT4b-2, 35S:PQT3-1, and 35S:PQT3-2 grown on MS with 150 μM CdCl2 for 14 days was measured. Values are mean ±SD (n = 30 plants, *P < 0.05). Asterisk indicate Student’s t-test significant difference. (E) The phenotype of wild type, 35S:PRMT4b-1, 35S:PRMT4b-2, 35S:PQT3-1, and 35S:PQT3-2 grown on MS medium with 150 mM NaCl for 14 days. Bar = 1 cm. (F) Primary root elongation of wild type, 35S:PRMT4b-1, 35S:PRMT4b-2, 35S:PQT3-1, and 35S:PQT3-2 grown on MS with 150 mM NaCl for 14 days was measured. Values are mean ± SD (n = 30 plants, *P < 0.05). Asterisk indicate Student’s t-test significant difference. (DOCX) [file pgen.1006332.s007.docx]

**Supporting Information for "PARAQUAT TOLERANCE3 is an E3 ligase that switches off activated oxidative response by targeting histone-modifying PROTEIN METHYLTRANSFERASE4b" by Luo et al.**


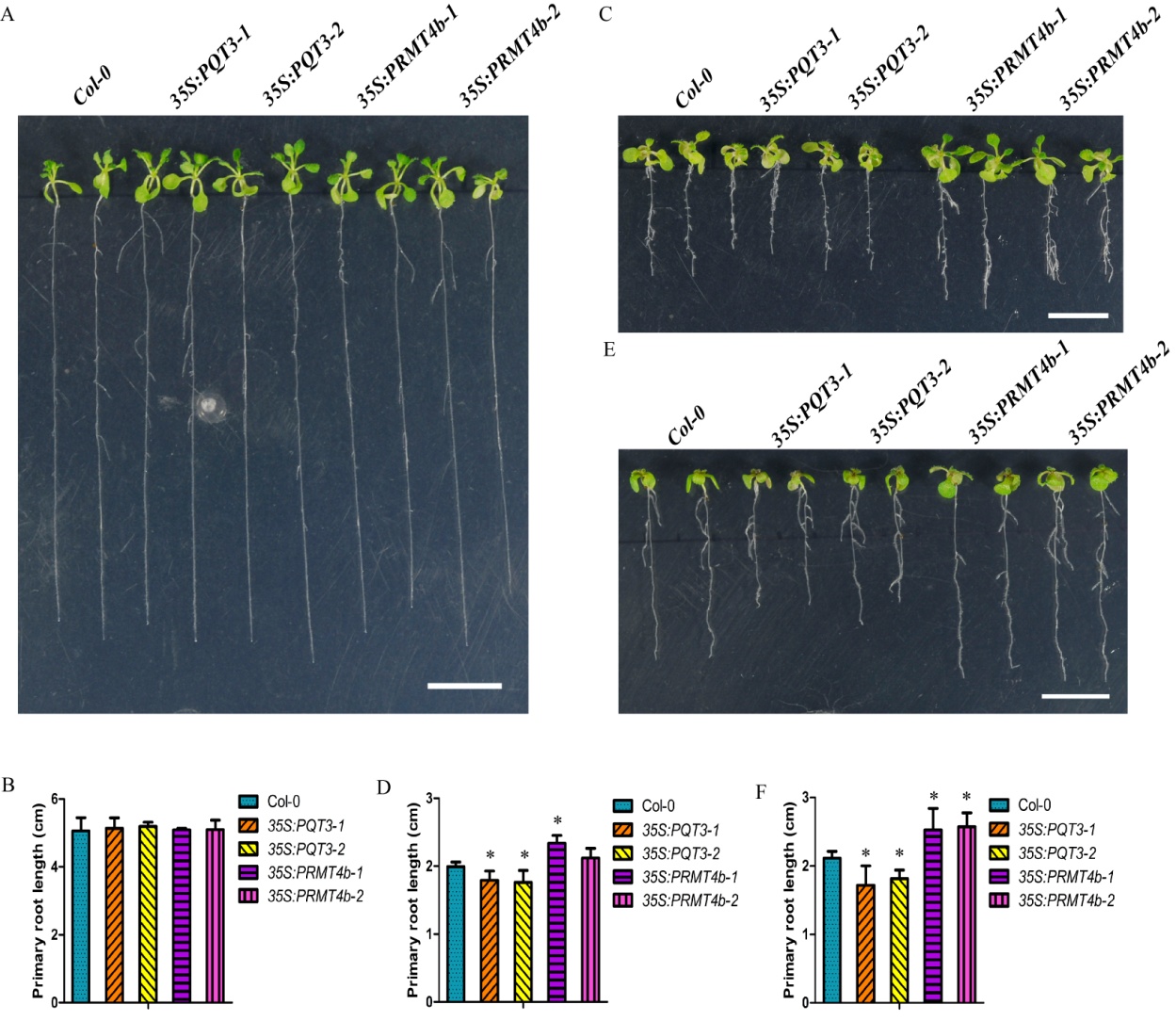


**S7 Fig. Phenotype of *PQT3* and *PRMT4b* overexpression lines under other environmental stresses lead to oxidative damage.**

**(A)** The phenotype of wild type, *35S:PRMT4b-1*, *35S:PRMT4b-2*, *35S:PQT3-1*, and *35S:PQT3-2* grown on MS medium for 14 days. Bar= 1 cm.

**(B)** Primary root elongation of wild type, *35S:PRMT4b-1*, *35S:PRMT4b-2*, *35S:PQT3-1*, and *35S:PQT3-2* grown on MS for 14 days was measured. Values are mean ± SD (n = 30 plants).

**(C)** The phenotype of wild type, *35S:PRMT4b-1*, *35S:PRMT4b-2*, *35S:PQT3-1*, and *35S:PQT3-2* grown on MS medium with 150 mM CdCl_2_ for 14 days. Bar= 1 cm.

**(D)** Primary root elongation of wild type, *35S:PRMT4b-1*, *35S:PRMT4b-2*, *35S:PQT3-1*, and *35S:PQT3-2* grown on MS with 150 mM CdCl_2_ for 14 days was measured. Values are mean ± SD (n = 30 plants, *P < 0.05). Asterisk indicates Student’s t-test signiﬁcant difference.

**(E)** The phenotype of wild type, *35S:PRMT4b-1*, *35S:PRMT4b-2*, *35S:PQT3-1*, and *35S:PQT3-2* grown on MS medium with 150 mM NaCl for 14 days. Bar= 1 cm.

**(F)** Primary root elongation of wild type, *35S:PRMT4b-1*, *35S:PRMT4b-2*, *35S:PQT3-1*, and *35S:PQT3-2* grown on MS with 150 mM NaCl for 14 days was measured. Values are mean ± SD (n = 30 plants, *P < 0.05). Asterisk indicates Student’s t-test signiﬁcant difference.
