# Supplementary material for: PARAQUAT TOLERANCE3 Is an E3 Ligase That Switches off Activated Oxidative Response by Targeting Histone-Modifying PROTEIN METHYLTRANSFERASE4b
Source: PLoS Genet. 2016 Sep 27;12(9):e1006332. doi: 10.1371/journal.pgen.1006332 (PMC5038976; doi:10.1371/journal.pgen.1006332)
Supplement: S8 Fig — (A-J) Quantitative RT-PCR analysis of transcript levels of antioxidant enzyme genes. RNA samples were isolated from 7-day-old wild type, prmt4b mutant and 35S:PRMT4b seedlings for quantitative RT-PCR analysis. The transcript levels of APX (A), GPX (B), ACHT (C), FSD (D), CAT (E), GRXC (F), CSD (G), PRXQ (H), 2CPB (I) and MSD (J) were analyzed. Values are mean ± SD (n = 3 experiments, *P < 0.05, **P < 0.01, ***P < 0.001). Asterisks indicate Student’s t-test significant differences. (DOCX) [file pgen.1006332.s008.docx]

**Supporting Information for "PARAQUAT TOLERANCE3 is an E3 ligase that switches off activated oxidative response by targeting histone-modifying PROTEIN METHYLTRANSFERASE4b" by Luo et al.**


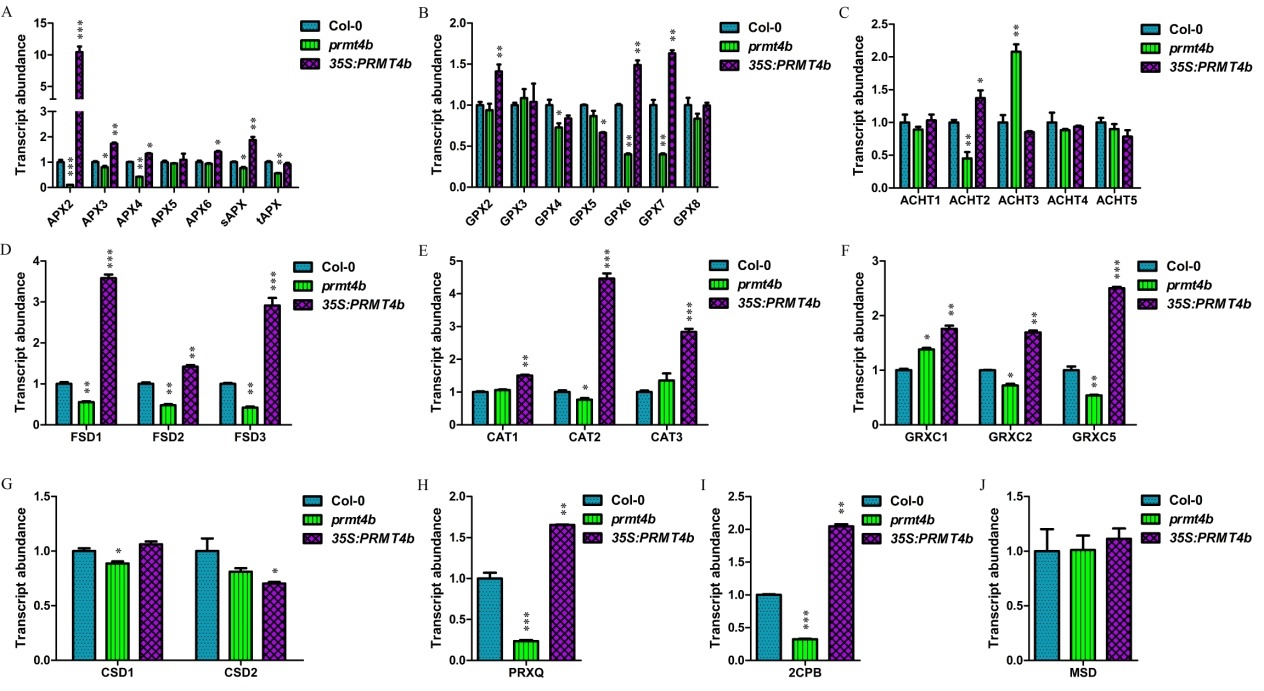


**S8 Fig. The analysis of antioxidant enzyme genes in wild type, *prmt4b* mutant, and *35Spro:PRMT4b*.**

**(A-J)** Quantitative RT-PCR analysis of transcript levels of antioxidant enzyme genes. RNA samples were isolated from 7-day-old wild type, *prmt4b* mutant and *35S:PRMT4b* seedlings for quantitative RT-PCR analysis. The transcript levels of APX **(A)**, GPX **(B)**, ACHT **(C)**, FSD **(D)**, CAT **(E)**, GRXC **(F)**, CSD **(G)**, PRXQ **(H)**, 2CPB **(I)** and MSD **(J)** were analyzed. Values are mean ± SD (n = 3 experiments, *P < 0.05, ***P < 0.01, ***P < 0.001). Asterisks indicate Student’s t-test signiﬁcant differences.
