# Supplementary material for: PARAQUAT TOLERANCE3 Is an E3 Ligase That Switches off Activated Oxidative Response by Targeting Histone-Modifying PROTEIN METHYLTRANSFERASE4b
Source: PLoS Genet. 2016 Sep 27;12(9):e1006332. doi: 10.1371/journal.pgen.1006332 (PMC5038976; doi:10.1371/journal.pgen.1006332)
Supplement: S9 Fig — (A) The conserved domains and NLSs (nuclear localization signals) of PQT3 protein. Based on predicted secondary structure with InterProScan protein sequence analysis software, the functional domains were marked using Vector NTI Advance software of Invitrogen. (B) Functional domains and the corresponding protein sequence of PQT3. Purple mark represents DWNN domain (amino acids 3–78); dark yellow mark represents Zinc finger (C2HC) domain (amino acids 201–218), and the red amino acids represent the conserved cysteine (C) and histidine (H); pink mark represents RING-finger (C6HC2) domain (amino acids 288–326), and the white amino acids represent the conserved cysteine (C) and histidine (H); yellow mark, including pink mark, represents U-box domain (amino acids 280–356); Two blue marks in C-terminus represent two NLS domains (amino acids 471–477 and 696–711). (C) Phylogenetic tree of PQT3 protein. AT4G17410: Arabidopsis thaliana PQT3; Bo_ABD64942.1, Bo_ABD65123.1: Brassica oleracea; Pt_XP_002326651.1: Populus trichocarpa; Rc_XP_002530663.1: Ricinus communis; Vv_CBI23464.3: Vitis vinifera; OsI_EEC67002.1: Oryza sativa Indica Group; OsJ_EEE50995.1: Oryza sativa Japonica Group. The result showed that the highest homology protein of Arabidopsis PQT3 is Bo_ABD65123.1 in Brassica oleracea. (DOCX) [file pgen.1006332.s009.docx]

**Supporting Information for "PARAQUAT TOLERANCE3 is an E3 ligase that switches off activated oxidative response by targeting histone-modifying PROTEIN METHYLTRANSFERASE4b" by Luo et al.**


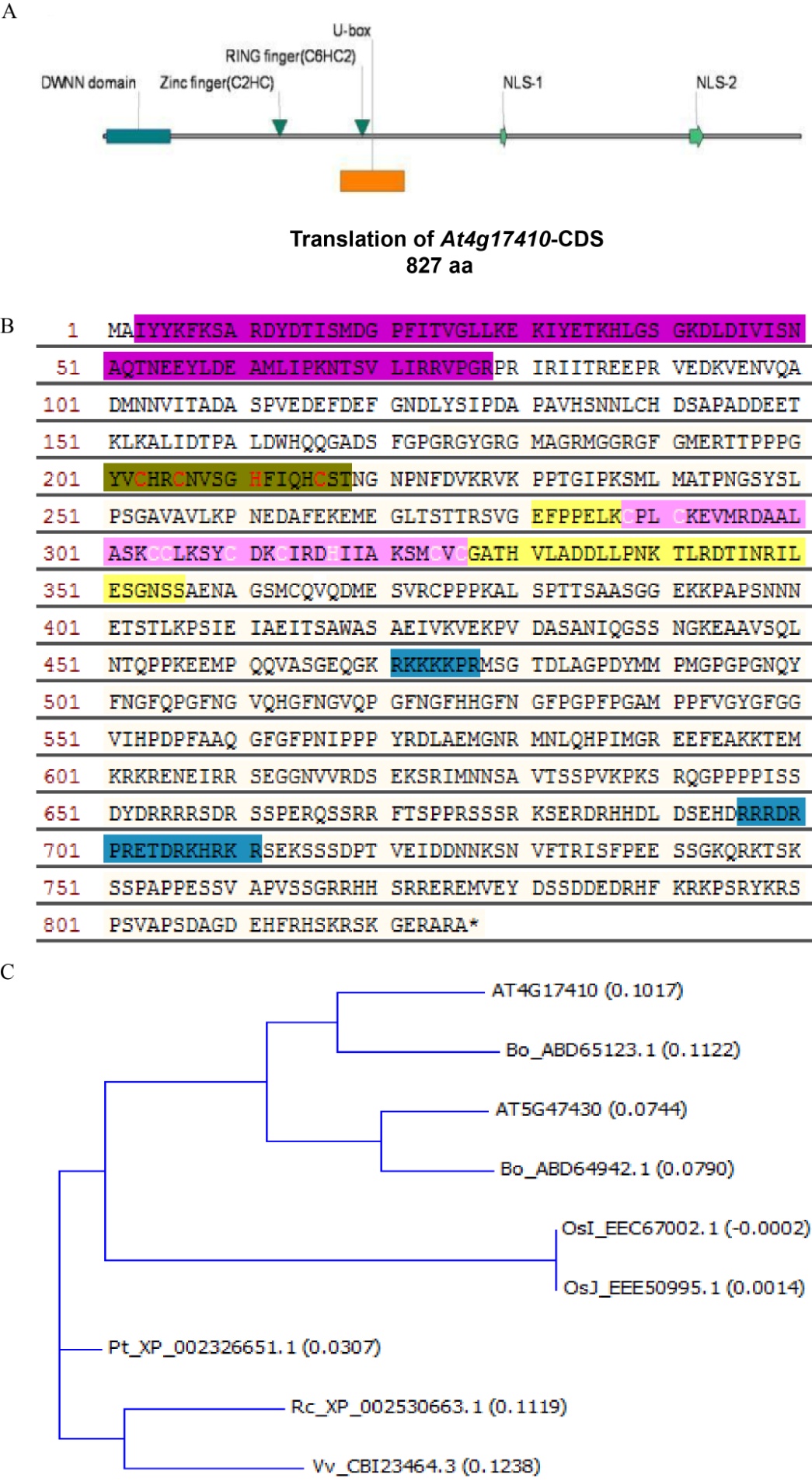


**S9 Fig. Functional domains and phylogenetic tree of PQT3 protein.**

**(A)** The conserved domains and NLSs (nuclear localization signals) of PQT3 protein. Based on predicted secondary structure with InterProScan protein sequence analysis software, the functional domains were marked using Vector NTI Advance software of Invitrogen.

**(B)** Functional domains and the corresponding protein sequence of PQT3. Purple mark represents DWNN domain (amino acids 3-78); dark yellow mark represents Zinc finger (C2HC) domain (amino acids 201-218), and the red amino acids represent the conserved cysteine (C) and histidine (H); pink mark represents RING-finger (C6HC2) domain (amino acids 288-326), and the white amino acids represent the conserved cysteine (C) and histidine (H); yellow mark, including pink mark, represents U-box domain (amino acids 280-356); Two blue marks in C-terminus represent two NLS domains (amino acids 471-477 and 696-711).

**(C)** Phylogenetic tree of PQT3 protein. AT4G17410: *Arabidopsis thaliana* PQT3; Bo_ABD64942.1, Bo_ABD65123.1: *Brassica oleracea*; Pt_XP_002326651.1: *Populus trichocarpa*; Rc_XP_002530663.1: *Ricinus communis*; Vv_CBI23464.3: *Vitis vinifera*; OsI_EEC67002.1: *Oryza sativa Indica Group*; OsJ_EEE50995.1: *Oryza sativa Japonica Group*. The result showed that the highest homology protein of Arabidopsis PQT3 is Bo_ABD65123.1 in *Brassica oleracea*.
