# Supplementary material for: PARAQUAT TOLERANCE3 Is an E3 Ligase That Switches off Activated Oxidative Response by Targeting Histone-Modifying PROTEIN METHYLTRANSFERASE4b
Source: PLoS Genet. 2016 Sep 27;12(9):e1006332. doi: 10.1371/journal.pgen.1006332 (PMC5038976; doi:10.1371/journal.pgen.1006332)
Supplement: S10 Fig — DWNN domain (3–78) and RING-finger/U-box domain (295–371) were conserved in different plants. Yellow shading indicates the same sequence of different plant proteins as PQT3; blue shading indicates the conserved sequence; green shading indicates the block similar sequence and white shading indicates the weak similar sequence (green word) and different sequence (black word). (DOCX) [file pgen.1006332.s010.docx]

**Supporting Information for "PARAQUAT TOLERANCE3 is an E3 ligase that switches off activated oxidative response by targeting histone-modifying PROTEIN METHYLTRANSFERASE4b" by Luo et al.**


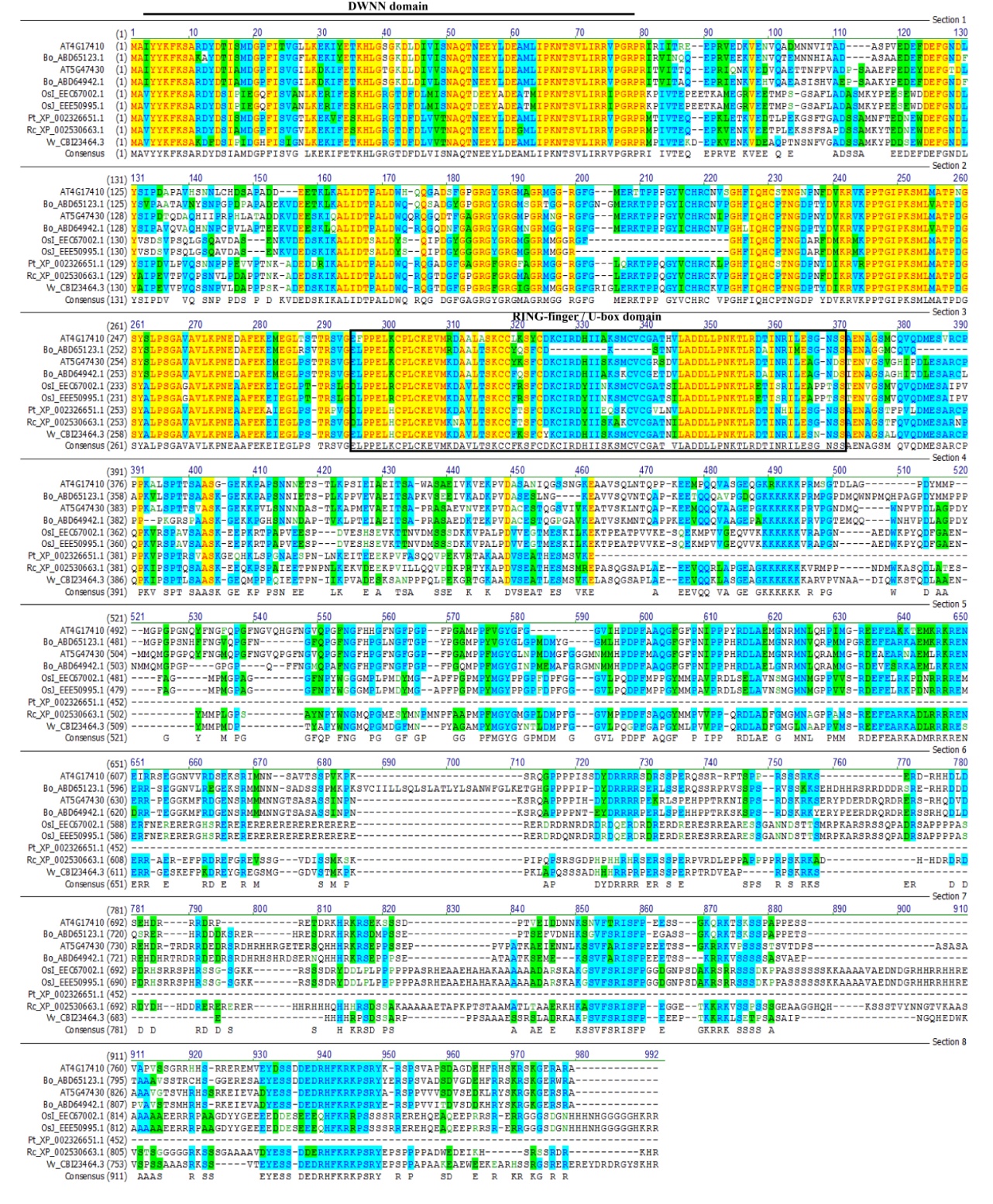


**S10 Fig. Homologous sequences alignment of PQT3 protein.**

DWNN domain (3-78) and RING-finger/U-box domain (295-371) were conserved in different plants. Yellow shading indicates the same sequence of different plant proteins as PQT3; blue shading indicates the conserved sequence; green shading indicates the block similar sequence and white shading indicates the weak similar sequence (green word) and different sequence (black word).
