# Supplementary material for: PARAQUAT TOLERANCE3 Is an E3 Ligase That Switches off Activated Oxidative Response by Targeting Histone-Modifying PROTEIN METHYLTRANSFERASE4b
Source: PLoS Genet. 2016 Sep 27;12(9):e1006332. doi: 10.1371/journal.pgen.1006332 (PMC5038976; doi:10.1371/journal.pgen.1006332)
Supplement: S1 Table — (DOCX) [file pgen.1006332.s011.docx]

**Supporting Information for "PARAQUAT TOLERANCE3 is an E3 ligase that switches off activated oxidative response by targeting histone-modifying PROTEIN METHYLTRANSFERASE4b" by Luo et al.**

**S1 Table. The primers used in this study.**

| GUS-PQT3 pro. LP | 5′-GTACAAAAAAGCAGGCTGCTAGGATCATAACCAAAAGG-3′ |
| --- | --- |
| GUS-PQT3 pro. RP | 5′-GTACAAGAAAGCTGGGTCGCAGAAACACGAACTCTAG-3′ |
| GFP-PQT3 P1 | 5′-GGGGACAAGTTTGTACAAAAAAGCAGGCTCG AGGTGAAGAGTCTTTTGGATTCG-3′ |
| GFP-PQT3 P2 | 5′-GGGGACCACTTTGTACAAGAAAGCTGGGTC AGCTCGCGCTCTTTCTCCTTTA-3′ |
| 8E-PQT3 LP | 5′-GACTAGTATGGCAATATATTACAAGTTTAAGAGTG-3′ |
| 8E-PQT3 RP | 5′-CCGCTCGAGAGCTCGCGCTCTTTCTCCTTTAG-3′ |
| GST-PQT3 CDS LP | 5′-GCGTCGACTGGCAATATATTACAAGTTTAAGAGT-3′ |
| GST-PQT3 CDS RP | 5′-AAGGAAAAAAGCGGCCGCTCAAGCTCGCGCTCTTTCTCC-3′ |
| GST-PQT3 N40 RP | 5′-AAGGAAAAAAGCGGCCGCTCAAGCGTTCTCAGCACTACTGTTA-3′ |
| MBP-PQT3-C66 P1 | 5′-GGaattccctaactttgatgttaagagagt-3′ |
| MBP-PQT3-C66 P2 | 5′-ACGCGTCGACtcaagctcgcgctctttctC-3′ |
| His-PRMT4b P1 | 5′-GGAATTCcatatggaggtatcttctgtgaaaaagct-3′ |
| His-PRMT4b P2 | 5′-CGGGATCCttagagctgggcacttgggtT-3′ |
| Y2H-PQT3 P1 | 5′-GGGGACAAGTTTGTACAAAAAAGCAGGCTCGATGGCAATATATTACAAGTTTAAGAGT-3′ |
| Y2H-PQT3 P2 | 5′-GGGGACCACTTTGTACAAGAAAGCTGGGTCTCAAGCTCGCGCTCTTTCTCCT-3′ |
| Y2H (PQT3) DWNN LP | 5′-TTGTACAAAAAAGCAGGCTCGATGGCAATATATTACAAGTTTAAGA-3′ |
| Y2H (PQT3) DWNN RP | 5′-TTTGTACAAGAAAGCTGGGTCTTAATCAGCTTGAACGTTTTCCACT-3′ |
| Y2H (PQT3) Znf C2HC LP | 5′-TTGTACAAAAAAGCAGGCTCGATGAACAATGTTATTACTGCTGAT-3′ |
| Y2H (PQT3) Znf C2HC RP | 5′-TTTGTACAAGAAAGCTGGGTCTTACAGCATCGACTTGGGGATAC-3′ |
| Y2H (PQT3) U-box LP | 5′-TTGTACAAAAAAGCAGGCTCGATGGCAACCCCAAATGGCT-3′ |
| Y2H (PQT3) U-box RP | 5′-TTTGTACAAGAAAGCTGGGTCTTAGCTGCCAGCGTTCTCAGCA-3′ |
| Y2H (PQT3) C-terminal LP | 5′-TTGTACAAAAAAGCAGGCTCGATGTGTCAAGTCCAAGATATGG-3′ |
| Y2H (PQT3) C-terminal RP | 5′-TTTGTACAAGAAAGCTGGGTCTCAAGCTCGCGCTCTTTCTC-3′ |
| Y2H-AtPRMT4b LP | 5′-TTGTACAAAAAAGCAGGCTCGATGGAGGTATCTTCTGTGAAAAA-3′ |
| Y2H-AtPRMT4b RP | 5′-TTTGTACAAGAAAGCTGGGTCTTAGAGCTGGGCACTTGGGT-3′ |
| Y2H /PRMT4a-p1 | 5′-GGGGACAAGTTTGTACAAAAAAGCAGGCTCG ATGGAGATTCCTTCTCTGAATAA-3′ |
| Y2H /PRMT4a-p2 | 5′-GGGGACCACTTTGTACAAGAAAGCTGGGTC CTAGAGCTGAGCGTTTGCGT-3′ |
| 35S PQT3 P1 | 5′-ggggacaagtttgtacaaaaaagcaggct atggcaatatattacaagtttaaga-3′ |
| 35S PQT3 P2 | 5′-ggggaccactttgtacaagaaagctgggt TCAAGCTCGCGCTCTTTCTC-3′ |
| 35S HA-PRMT4b P1 | 5′-ggggacaagtttgtacaaaaaagcaggct atgtacccatacgatgttccagat tacgctATGGAGGTATCTTCTGTGAAAA-3′ |
| 35S HA-PRMT4b P2 | 5′-ggggaccactttgtacaagaaagctgggt TTAGAGCTGGGCACTTGGGT-3′ |
| 35S PRMT4b P1 | 5′-ATGGAGGTATCTTCTGTGAAA-3′ |
| 35S PRMT4b P2 | 5′-TTAGAGCTGGGCACTTGGG-3′ |
| PRMT4b Q-PCR P1 | 5′-TGCCAAACATGTGTATGCGGTT-3′ |
| PRMT4b Q-PCR P2 | 5′-ACCTTGGTGCGCTGATCCATA-3′ |
| APX1(ChIP) -A-P1 | 5′-taccaccaaccctaagatccga-3′ |
| APX1(ChIP) -A-P2 | 5′-aatgatccgatcgagtcaagatga-3′ |
| APX1(ChIP) –B-P1 | 5′-gatcggcgttattatcgtcagc-3′ |
| APX1(ChIP) –B-P2 | 5′-gttgggtagttcttcgtcatctt-3′ |
| APX1(ChIP) –C-P1 | 5′-AGTAACTAACCATATTTGCTTGTG-3′ |
| APX1(ChIP) –C-P2 | 5′-CAAAAGAGATGGTAGGGAATTG-3′ |
| APX1(ChIP) –D-P1 | 5′-cattcagcttgctggtgttgtg-3′ |
| APX1(ChIP) –D-P2 | 5′-caccagataaagcgacaatgtct-3′ |
| APX1(ChIP) –E-P1 | 5′-CAGGGACGATGCCACAAGGA-3′ |
| APX1(ChIP) –E-P2 | 5′-ACATACAGCAGCGTATTTCTCG-3′ |
| APX1(ChIP) –F-P1 | 5′-tctgtttttcacttggtagagaga-3′ |
| APX1(ChIP) –F-P2 | 5′-CAACCAAAAACAGCCATGACTCT-3′ |
| GPX1(ChIP) -A-P1 | 5′-cttgagatgtgtaatctttacagc-3′ |
| GPX1(ChIP) -A-P2 | 5′-ctattacttcacatacctaagtgc-3′ |
| GPX1(ChIP) –B-P1 | 5′-gttttgagaggcttgaagcacaa-3′ |
| GPX1(ChIP) –B-P2 | 5′-CCCAATCAAAAAGTGACATGTAAGT-3′ |
| GPX1(ChIP) –C-P1 | 5′-tcggaataacgagtgggagtga-3′ |
| GPX1(ChIP) –C-P2 | 5′-ATCTAACCGGTGGCAATGCCTA-3′ |
| GPX1(ChIP) –D-P1 | 5′-gaggatgaaacatagtccaaaaca-3′ |
| GPX1(ChIP) –D-P2 | 5′-ctgcagtattttctatgatttatgg-3′ |
| GPX1(ChIP) –E-P1 | 5′-tttcatagaataaaatgctccagaat-3′ |
| GPX1(ChIP) –E-P2 | 5′-gagatgatatagtcgagacacga-3′ |
| GPX1(ChIP) –F-P1 | 5′-cgaacataaactcgtctccggt-3′ |
| GPX1(ChIP) –F-P2 | 5′-agaaccaaaccttaacggtgaaat-3′ |
| GPX1(ChIP)-G-P1 | 5′-ggaatcttcaactttgcaatgaag-3′ |
| GPX1(ChIP)-G-P2 | 5′-taccatcttgaagcaacattgaca-3′ |
| GPX1(ChIP)-H-P1 | 5′-tcagagctttcacatctgtacga-3′ |
| GPX1(ChIP)-H-P2 | 5′-tacagagaacggatgagtgagat-3′ |
| GPX1(ChIP)-I-P1 | 5′-tctagcttttccctgcaatcagt-3′ |
| GPX1(ChIP)-I-P2 | 5′-cgatttggaaaggggatgtggt-3′ |
| PQT3 Q-PCR P1 | 5′-tacttctgtgctaattcgccgggt-3′ |
| PQT3 Q-PCR P2 | 5′-GGACTGCTGGGGCATCAGGAATT-3′ |
| BiFC /PRMT4b P1 | 5′-CCATCGATatggaggtatcttctgtgaaAAA-3′ |
| BiFC /PRMT4b P2 | 5′-CCGCTCGAGgagctgggcacttgggtTCT-3′ |
| BiFC /PQT3 P1 | 5′-CCATCGATATGGCAATATATTACAAGTTTAAGA-3′ |
| BiFC /PQT3 P2 | 5′-CCGCTCGAGagctcgcgctctttctCCTT-3′ |
| β－tubulin8 P1 | 5′-CTTAAGCTCACCACTCCAAGCT-3′ |
| β－tubulin8 P2 | 5′-GCACTTCCACTTCGTCTTCTTC-3′ |
| APX1 Q-PCR LP | 5′-gtccgactcgcatggcactc-3′ |
| APX1 Q-PCR RP | 5′-acaacaccagcaagctgatgga-3′ |
| APX2 Q-PCR LP | 5′-ATTGTCTGGTGGACACACCTTGGG-3′ |
| APX2 Q-PCR RP | 5′-ATGCACCCTCGAATCCTGAACG-3′ |
| APX3 Q-PCR LP | 5′-AGAGCACACTCATGGTGCCAAC-3′ |
| APX3 Q-PCR RP | 5′-TGCTTAGCTTTCACGCCCTCAC-3′ |
| APX4 Q-PCR LP | 5′-GCATATGGTTCAGCTGGTCAGTGG-3′ |
| APX4 Q-PCR RP | 5′-TCAGCCTCTGTTGCATCACTCC-3′ |
| APX5 Q-PCR LP | 5′-AGCATCCCAGAGTCTCTTACGC-3′ |
| APX5 Q-PCR RP | 5′-TCCGCGGAATCAGCATCCTTAC-3′ |
| APX6 Q-PCR LP | 5′-TATGTGGCGGCCCAACAATTCC-3′ |
| APX6 Q-PCR RP | 5′-TTGCCTTCTGGATCAGGTTGCG-3′ |
| sAPX Q-PCR LP | 5′-TCTAGGCCAGAACGTAGTGGTTGG-3′ |
| sAPX Q-PCR RP | 5′-TGCTCCAGGTCCTTCTTTCGTG-3′ |
| tAPX Q-PCR LP | 5′-GCTAGTGCCACAGCAATAGAGGAG-3′ |
| tAPX Q-PCR RP | 5′-TGATCAGCTGGTGAAGGAGGTC-3′ |
| CSD1Q-PCR-LP | 5′-AACGGTTGCATGTCTACTGGTC-3′ |
| CSD1Q-PCR-RP | 5′-GTGATTGTGAAGGTGGCAGTTCC-3′ |
| CSD2Q-PCR-LP | 5′-CAGGGCCTCATGGATTTCATCTCC-3′ |
| CSD2Q-PCR-RP | 5′-TGGAGCTCCGTGTGTCATGTTG-3′ |
| CAT1 Q-PCR-LP | 5′-TCGGGAAGGAGAACAACTTCAAGC-3′ |
| CAT1 Q-PCR-RP | 5′-TCACGAATCGTTCTTGCCTGTC-3′ |
| CAT2 Q-PCR-LP | 5′-AAGTATCCAACTCCGCCTGCTG-3′ |
| CAT2 Q-PCR-RP | 5′-TGGATGAATCGTTCTTGCCTCTC-3′ |
| CAT3 Q-PCR-LP | 5′-AGGTACAGATCATGGGCACCAG-3′ |
| CAT3 Q-PCR-RP | 5′-AAGGATCGATCAGCCTGAGACC-3′ |
| FSD1 Q-PCR-LP | 5′-AGTGCTGTCACCGCAAACTACG-3′ |
| FSD1 Q-PCR-RP | 5′-TATGCGGCTCCAAAGCATCCAG-3′ |
| FSD2 Q-PCR-LP | 5′-GGTGGCTGTTTCCGGTGTTATC-3′ |
| FSD2 Q-PCR-RP | 5′-TATGCGGTTCCAGAGCATCAAG-3′ |
| FSD3 Q-PCR-LP | 5′-CCACTCGTGTGGGACGATATTC-3′ |
| FSD3 Q-PCR-RP | 5′-ACGACACCAAGTGGTTCAGAAATG-3′ |
| GPX1 Q-PCR-LP | 5′-gttgattgtcaatgttgcttcaag-3′ |
| GPX1 Q-PCR-RP | 5′-ATAGGGAACTCTGCTTTAAACCG-3′ |
| GPX2 Q-PCR-LP | 5′-TCCTGATGGCAAGGTCTTACAGAG-3′ |
| GPX2 Q-PCR-RP | 5′-ACGCAGTTTGAATGTCCTTCTCG-3′ |
| GPX3 Q-PCR-LP | 5′-CCATCGACGGTGGAACAATCATCC-3′ |
| GPX3 Q-PCR-RP | 5′-GCACTTTCCCGGTAAACTTGCTC-3′ |
| GPX4 Q-PCR-LP | 5′-TCGTCAATGTTGCTTCCAAATGCG-3′ |
| GPX4 Q-PCR-RP | 5′-ACTGGTTGCAAGGGAATGCCAAG-3′ |
| GPX5 Q-PCR-LP | 5′-TTGGTCGGCAAAGATGGTCAAG-3′ |
| GPX5 Q-PCR-RP | 5′-AGGGCTTTCTCGATGTCTTTCTGG-3′ |
| GPX6 Q-PCR-LP | 5′-CAATGGCTGCTTCTTCCGAACC-3′ |
| GPX6 Q-PCR-RP | 5′-ACATCGTTTCCCTTAGCATCCTTG-3′ |
| GPX7 Q-PCR-LP | 5′-CGTTAACGTTGCGTCAAGATGTGG-3′ |
| GPX7 Q-PCR-RP | 5′-TGACCTCCAAATTGATTGCAAGGG-3′ |
| GPX8 Q-PCR-LP | 5′-TGTTGCTTCCAAATGTGGGATGAC-3′ |
| GPX8 Q-PCR-RP | 5′-TTAGTTCCCGGTTCCTCGTCAC-3′ |
| ACHT1 Q-PCR-LP | 5′-TGCCGTGCAATGTTCCCAAAGC-3′ |
| ACHT1 Q-PCR-RP | 5′-CACCGCGGTAGAAGTGGAAATACG-3′ |
| ACHT2 Q-PCR-LP | 5′-GCCAAGTGGTTCGTGTCAAGGTAG-3′ |
| ACHT2 Q-PCR-RP | 5′-AGCATTCCTCTCCCACCATTTCG-3′ |
| ACHT3 Q-PCR-LP | 5′-GCAACCATTTCAGCCCGAGAAC-3′ |
| ACHT3 Q-PCR-RP | 5′-TCCCAAGACTTGTCTGACATCGG-3′ |
| ACHT4 Q-PCR-LP | 5′-TCGGAGATTCTCAAGACGAGAGC-3′ |
| ACHT4 Q-PCR-RP | 5′-CCCAATCCTCAAAGTTGTCTGAGC-3′ |
| ACHT5 Q-PCR-LP | 5′-AGCGTTGGATAAGCACGGTAGTG-3′ |
| ACHT5 Q-PCR-RP | 5′-TCTCGTCTAATCCCTTTGCATCCC-3′ |
| 2CPB Q-PCR-LP | 5′-TTCTCTCTGTTCCGGGTTCTCCAG-3′ |
| 2CPB Q-PCR-RP | 5′-ACCGACCAGTGGTAAATCATCAGC-3′ |
| PRXQ Q-PCR-LP | 5′-AGATGACTCTGCTTCTCACAAGGC-3′ |
| PRXQ Q-PCR-RP | 5′-TCCCTGGCAATGCTCCAAACAG-3′ |
| GRXC1 Q-PCR-LP | 5′-GCTTATCCCGTTGTTGTCTTCAGC-3′ |
| GRXC1 Q-PCR-RP | 5′-AGCTGCGTCAGTAACTGCTTCAC-3′ |
| GRXC2 Q-PCR-LP | 5′-ATCACATCGGTGGCTGTGATGC-3′ |
| GRXC2 Q-PCR-RP | 5′-ACAGCGGAACCAACTTCCCATC-3′ |
| GRXC5 Q-PCR-LP | 5′-ACATTGGTGGCTGCACAGATACAG-3′ |
| GRXC5 Q-PCR-RP | 5′-ACCGTTGGCTTCAGCTAACATCAG-3′ |
| MSD (Q-PCR) P1 | 5′-ggaggtcatgtcaaccattcga-3′ |
| MSD (Q-PCR) P2 | 5′-cttcagttctttgtctagtccga-3′ |
| *UBQ5*(Q-PCR) P1 | 5′-AGAAGATCAAGCACAAGCAT-3′ |
| *UBQ5*(Q-PCR) P2 | 5′-CAGATCAAGCTTCAACTCCT-3′ |
| Salk_097442(*prmt4b*)-LP | 5′-CACGGGCTGTAAAATCCATAG-3′ |
| Salk_097442(*prmt4b*)-RP | 5′-CTCTCCTTGCCTTAATCCACC-3′ |
| Salk_033423 (*prmt4a*)-P1 | 5′-CGTCCCTGTTTAGTGAATGGCA-3′ |
| Salk_033423 (*prmt4a*)-P2 | 5′-GCTGAGCGTTTGCGTTTTGTT-3′ |
| LBb1.3 | 5′-ATTTTGCCGATTTCGGAAC-3′ |
| PQT3 PT-PCR-P1 | 5′-TGACATTGTCATCTCTAATGCC-3′ |
| PQT3 RT-PCR-P2 | 5′-GCGTTCTCAGCACTACTGTTA-3′ |
| Salk_065409(*pqt3-2*)-LP | 5′-TCGTATGAATCTCCAGCATCC-3′ |
| Salk_065409(*pqt3-2*)-RP | 5′-CGTGGATTACAAACGTGTTTTG-3′ |
